# Phospholipase D2 acts upstream of PI4P 5-kinases in the response of neurons to extracellular viscosity

**DOI:** 10.1016/j.isci.2026.117267

**Published:** 2026-08-24

**Authors:** Trisha Kundu, Andreas W. Püschel

**Affiliations:** 1 Institut für Integrative Zellbiologie und Physiologie, Universität Münster, Schloßplatz 5, 48149 Münster, Germany; 2 Cells-in-Motion Interfaculty Center, University of Münster, 48149 Münster, Germany

**Keywords:** neuronal polarity, axon extension, phospholipase D, phospholipids, extracellular viscosity, gangliosides, GM1

## Abstract

The formation of an axon during neuronal differentiation is determined by the competition of positive and negative signals that promote and inhibit extension, respectively. Several biochemical signals have been identified that promote axon extension, but it is just beginning to be appreciated that the physical properties of the extracellular environment also play an important role. Using primary cultures, we show that developing central neurons respond to an increase in extracellular viscosity. This response depends on plasma membrane glycolipids and Phospholipase D2 (Pld2). Pld2 acts upstream of type 1 phosphatidylinositol 4-phosphate 5-kinases (Pip5ks) to regulate the generation of phosphatidylinositol-4,5-bisphosphate (PI(4,5)P_2_) as an inhibitory signal for axon extension. The glucosylceramide GM1 that negatively regulates Pld2 is increased in neurites by a shift to high viscosity. Our results suggest that changes in the physical properties of the extracellular environment regulate axon extension by modulating plasma membrane glycolipids and Pld2/Pip5k1 signaling.

## Highlights

• Neurons are sensitive to an increase in extracellular viscosity
• The response to high viscosity depends on Pld2 upstream of Pip5ks
• A shift to high viscosity reduces Pl(4,5)P_2_ levels
• The response to high viscosity requires VLCFA and gangliosides

## Graphical abstract

**Figure:**
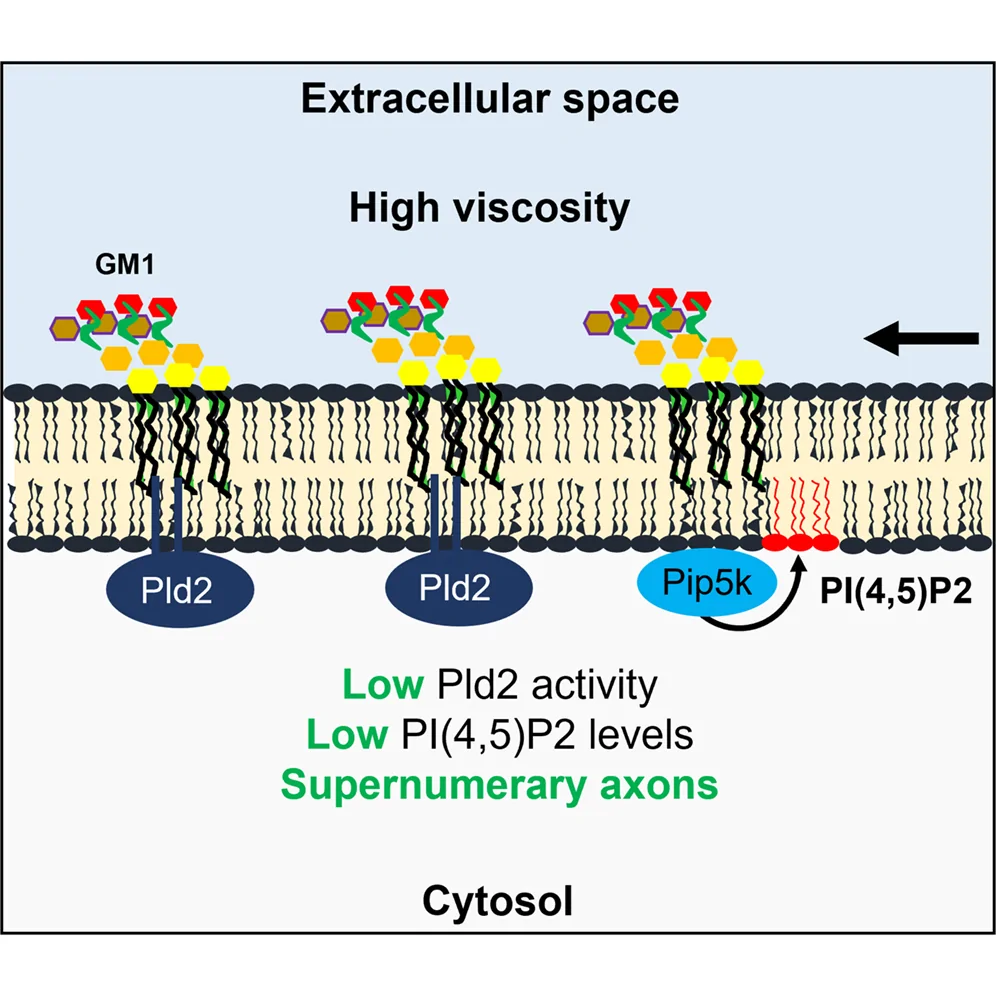

## Teaser

Molecular biology; Molecular interaction; Molecular neuroscience

## Introduction

Newborn neurons initially form several neurites that have the potential to become an axon.^1^^,^^2^^,^^3^ This initial, unpolarized state is maintained as long as positive and negative signals that promote and inhibit axon extension, respectively, are balanced.^3^^,^^4^ Axon extension is initiated when, in one of the neurites, the growth promoting signals become stronger than the inhibitory ones. This happens when neurons migrate into the intermediate zone (IZ) after their generation in the ventricular and subventricular zone of the developing cortex.^1^^,^^2^

The IZ is rich in extracellular matrix (ECM) that is composed of multiple proteoglycans and glycosaminoglycans.^5^ An important component is hyaluronic acid (HA) as one of the major glycosaminoglycans in the nervous system.^6^ Interfering with its function by digestion of HA or disruption of HA-binding proteins impairs the polarization and migration of neurons.^6^ The components of the ECM can act as biochemical cues but also determine the physical properties of the extracellular environment that migrating neurons encounter.^7^^,^^8^^,^^9^ High concentration of HA suggests that the extracellular space of the IZ displays a higher viscosity compared to other cortical layers.^10^ Recent results show that a high viscosity can promote cell migration but its role during neuronal differentiation remains to be elucidated.^11^^,^^12^

We have shown previously that the generation of PI(4,5)P_2_ is required in developing central neurons as an inhibitory signal that ensures the formation of a single axon.^13^ PI(4,5)P_2_ is mainly synthesized by Pip5ks.^9^^,^^14^ Knockdown or inhibition of each of the three Pip5ks (Pip5k1α, Pip5k1β, or Pip5k1χ) results in the formation of supernumerary axons.^13^ Shifting unpolarized neurons to hyperosmotic medium to modulate membrane tension has the same effect, which can be reversed by expression of constitutively active Pip5k1α. These results showed that PI(4,5)P_2_ acts as an inhibitory signal that limits the potential to extend as an axon to a single neurite but it remained to be elucidated how Pip5ks are regulated.

The catalytic activity of Pip5ks is strongly stimulated by phosphatidic acid (PA).^9^^,^^14^ The production of PA at the plasma membrane is driven by Phospholipase D1 (Pld1) and Pld2 that convert phosphatidylcholine to PA.^15^^,^^16^ Both isoforms are expressed in neurons and have been linked to neurite outgrowth.^15^^,^^17^ Pld2 has been implicated in mediating the cellular response to mechanical stimuli and changes in membrane tension as osmotic shock or shear stress regulate its activity.^18^^,^^19^^,^^20^^,^^21^ The activation of Pld2 by shear stress results from disrupting its association with membrane domains containing the ganglioside GM1.^18^

Here, we show that neurons are sensitive to changes in extracellular viscosity. A shift to high viscosity induces the formation of supernumerary axons, indicating that it reduces an inhibitory signal. Expression of constitutively active Pld2 or Pip5k1α prevents this effect. Pld2 acts upstream of Pip5ks to regulate PI(4,5)P_2_ production and axon extension. Very-long-chain fatty acids (VLCFAs) and glucosylceramides are required for the response to high viscosity as inhibiting their synthesis prevents the induction of supernumerary axons. Our results suggest that the response of neurons to extracellular viscosity is mediated by plasma membrane glycolipids and Pld2/Pip5k signaling that regulates axon formation.

## Results

### A shift to high viscosity induces supernumerary axons

**Figure 1:**
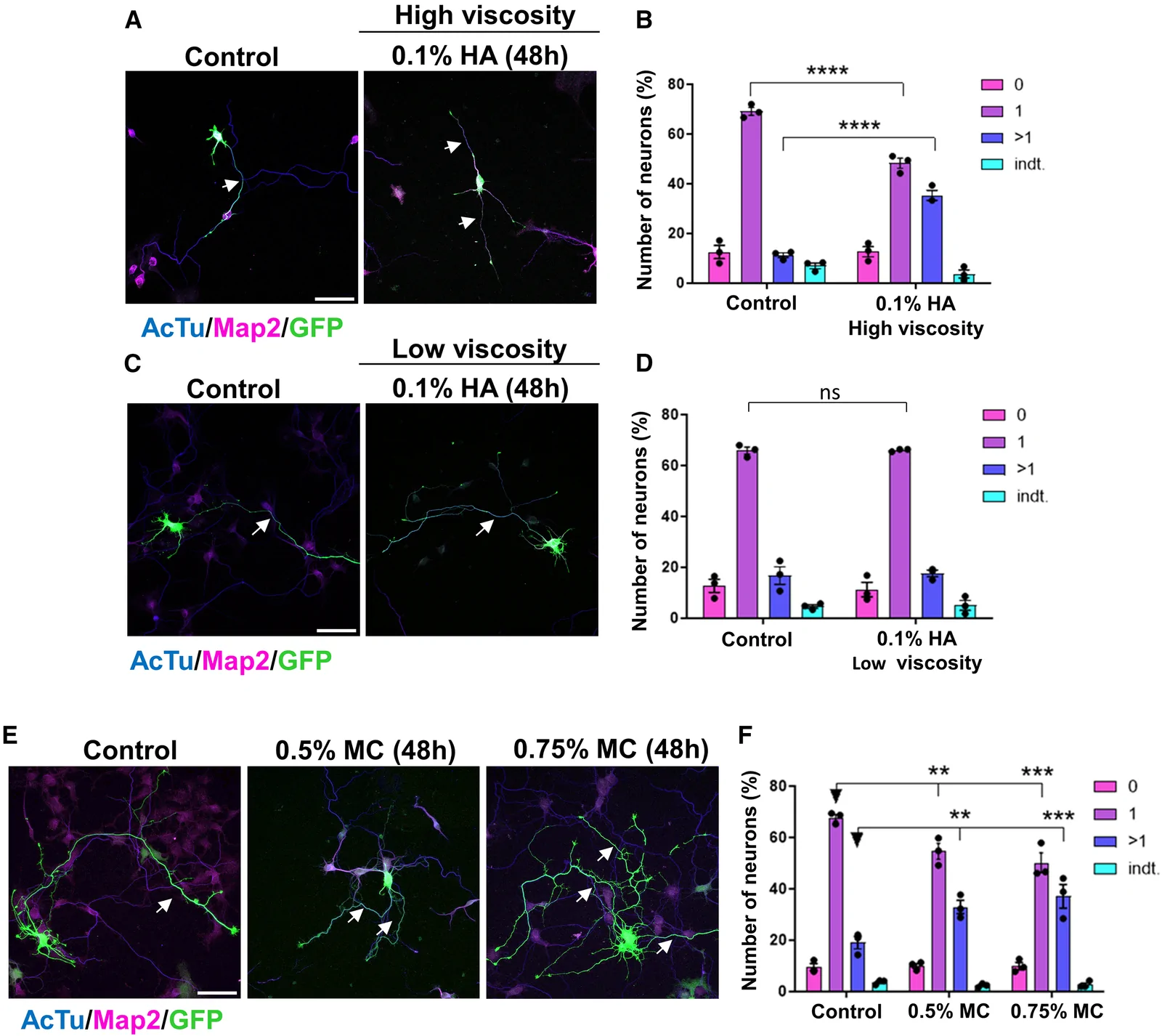
A shift to high viscosity induces supernumerary axons (A and C) Hippocampal neurons were transfected with a vector for GFP (green) and shifted to medium supplemented with 0.1% high viscosity HA (A) or 0.1% low viscosity HA (C) at 1 d.i.v. for 48 h. Neurons were analyzed at 3 d.i.v. by staining with anti-acetylated tubulin (axons, blue) and anti-Map2 (minor neurites, magenta) antibodies. Axons are marked by arrows. Scale bars, 50 μm. (B and D) The percentage of unpolarized neurons without an axon (magenta, 0), polarized neurons with a single axon (purple, 1) and neurons with supernumerary axons (blue, >1) or indeterminate neurites (cyan, indt.) was quantified for (A) and (C). (E) Hippocampal neurons were transfected with a vector for GFP (green), shifted to medium supplemented with 0.5% and 0.75% MC at 1 d.i.v. for 48 h and analyzed at 3 d.i.v. as in (A). Scale bars, 50 μm. (F) Axon formation was quantified for (E) as in (B). See also Figure S1. Values are means ± SEM from *n* = 3 independent experiments that are biological replicates. (B, D, and F) Two-way ANOVA followed by Tukey’s post hoc test. *p* > 0.05; ∗*p* ≤ 0.05; ∗∗*p* ≤ 0.01; ∗∗∗*p* ≤ 0.001; ∗∗∗∗*p* ≤ 0.0001.

Viscosity is an important property of the extracellular environment that regulates cell migration.^11^^,^^12^ To determine if neurons respond to a change in viscosity, we tested if it affects axon extension. Primary hippocampal neurons were exposed at 1 d.i.v. to medium supplemented with 0.1% high-molecular-weight (MW) HA, which has a viscosity of approximately 3.7 cP,^22^ compared to 0.77 cP for normal medium.^11^ Axon formation was analyzed at 3 d.i.v. by staining with antibodies specific for axons (acetylated tubulin) and minor neurites (MAP2).^23^^,^^24^ When neurons were shifted to medium with 0.1% high viscosity HA, the percentage that extended supernumerary axons increased (35% ± 2% supernumerary neurons) compared to normal, low viscosity medium (11% ± 1% supernumerary neurons; Figures 1A and 1B). The viscosity of an HA solution depends on its MW.^10^ A shift to 0.1% low MW HA with low viscosity did not significantly increase the number of neurons with supernumerary axons (control: 17% ± 3%, low viscosity HA: 18% ± 1%; Figures 1C and 1D).

To determine if the chemical nature of the polymer is relevant, the effects of hydroxypropyl methylcellulose (MC) were tested.^12^ When neurons were shifted to a high viscosity medium with 0.5% or 0.75% MC at 1 d.i.v. and axon formation was analyzed at 3 d.i.v., the percentage of neurons with multiple axons increased compared to control (control: 19% ± 2% supernumerary axons, 0.5% MC: 33% ± 3%, 0.75% MC: 37% ± 5%; Figures 1E and 1F). No significant change in the length of the axons was observed (control: 386 ± 23 μm, 0.5% MC: 428 ± 22 μm, 0.75% MC: 398 ± 21 μm; Figure S1A). The addition of MC did not increase the osmolarity of the medium (control medium: 217 ± 2 mOsm/L, 0.5% MC: 216 ± 2 mOsm/L, 0.75% MC: 222 ± 3 mOsm/L). Since 0.75% MC with a viscosity of 4.99 cP had the most robust effect without compromising neuronal viability, this concentration was chosen for the further experiments.

To confirm that a shift to high viscosity medium induces the formation of supernumerary axons, neurons were shifted to medium with 0.75% MC at 1 d.i.v. for 48 or 96 h and axon extension was analyzed using the Tau-1 antibody as an additional axonal marker.^25^ The percentage of neurons with multiple axons that are positive for Tau-1 staining was increased at 3 d.i.v. (control: 15% ± 3% supernumerary axons, 0.75% MC: 33% ± 1%; Figures S1B and S1C) and 5 d.i.v. (control: 18% ± 2% supernumerary axons, 0.75% MC: 33% ± 2%; Figures S1D and S1E). These results show that unpolarized neurons respond to an increased viscosity with the formation of supernumerary axons.

### Pld1 and Pld2 are required for the establishment of neuronal polarity

Pld2 has been identified as one of the factors that mediate the response to physical stimuli.^18^^,^^19^^,^^21^ To determine if Plds are involved in the response to high viscosity, we first investigated their role during axon formation with knockdown vectors targeting Pld1 or Pld2. The efficiency of the RNAi constructs was confirmed in HEK293T cells by western blot (Figures S2A, S2B, S2E, and S2F) and staining neurons with antibodies against Pld1 or Pld2 (Figures S2C, S2D, S2G, and S2H).

**Figure 2:**
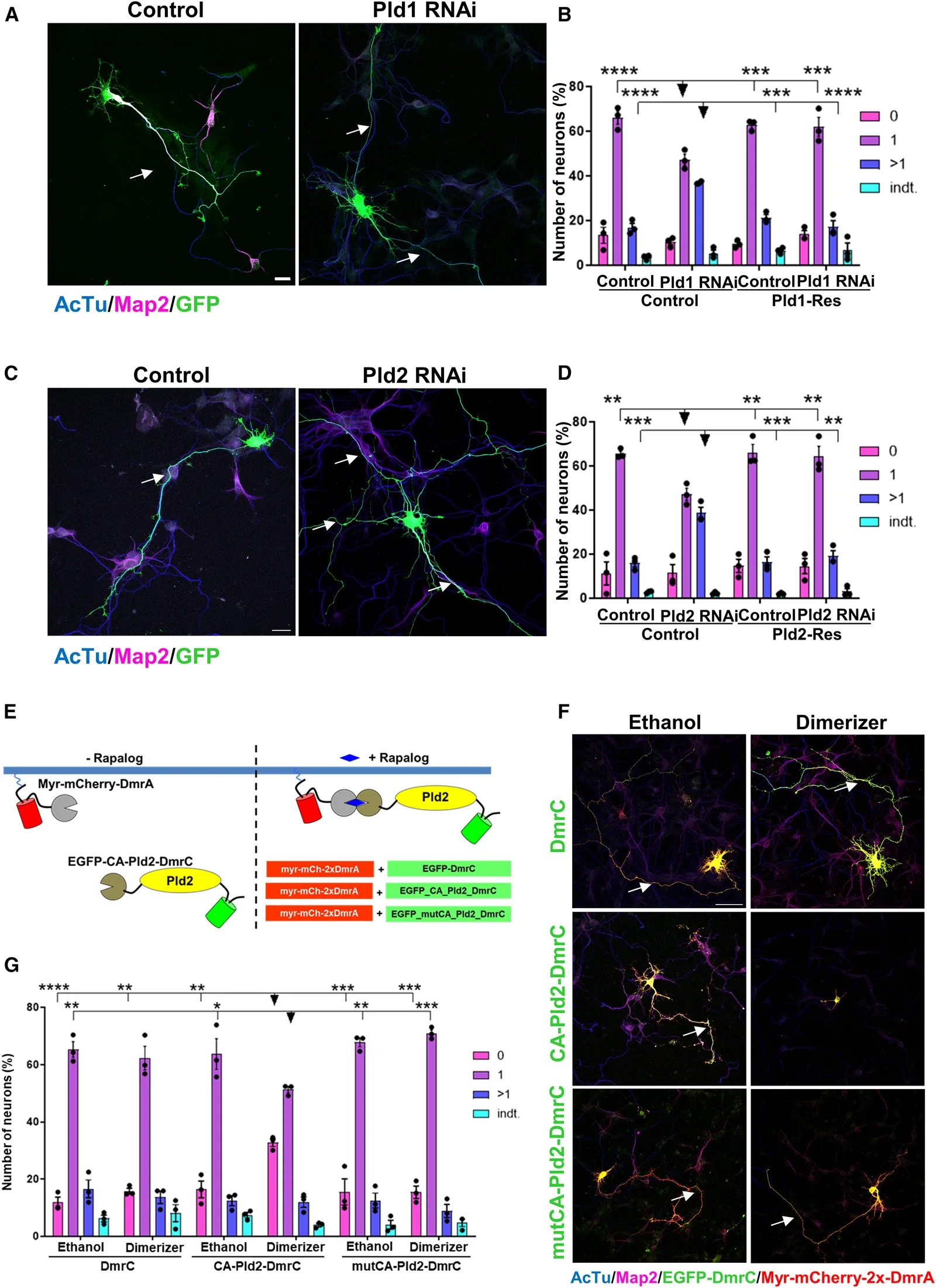
Plds are required for the establishment of neuronal polarity (A and C) Hippocampal neurons were transfected with control or knockdown vectors directed against Pld1(A) or Pld2 (C) and analyzed at 3 d.i.v. by staining with anti-acetylated tubulin (blue) and anti-Map2 (magenta) antibodies. Axons are marked by arrows. Scale bars, 20 μm. (B and D) The percentage of neurons without axons (0, magenta), with a single axon (1, purple), with supernumerary axons (>1, blue) and with indeterminate axons positive for both acetylated tubulin and Map2 (indt., cyan) was quantified for (A) and (C). (E) Rapalog-induced heterodimerization was used to activate Pld2 by recruitment to the plasma membrane. Truncated Pld2 was fused to DmrC and EGFP (EGFP-CA-Pld2-DmrC) and DmrA with an N-terminal myristoylation signal and mCherry (myr-mCherry-2x-DmrA). (F) Cortical neurons were transfected with vectors for myr-mCherry-2x-DmrA (red) and EGFP-DmrC (control), EGFP-CA-Pld2-DmrC or EGFP-mutCA-Pld2-DmrC (inactive Pld2). Solvent (control: ethanol) or dimerizer was added at 1 d.i.v. for 30 min. Axon formation was analyzed at 3 d.i.v. as in (A). Scale bars, 50 μm. (G) Axon extension was quantified for (F) as in (B). See also Figures S2 and S3. Values are means ± SEM from *n* = 3 independent experiments that are biological replicates. (B, D, and G) Two-way ANOVA followed by Tukey’s post hoc test. *p* > 0.05; ∗*p* ≤ 0.05; ∗∗*p* ≤ 0.01; ∗∗∗*p* ≤ 0.001; ∗∗∗∗*p* ≤ 0.0001.

Hippocampal neurons were transfected with the knockdown vectors at 0 d.i.v. and axon formation was analyzed at 3 d.i.v. (Figure 2). The knockdown of Pld1 (control: 17% ± 2% supernumerary axons, Pld1 RNAi: 37% ± 0.3%; Figures 2A and 2B) or Pld2 (control: 16% ± 2%, Pld2 RNAi: 39% ± 3%; Figures 2C and 2D) resulted in an increase in the number of neurons with supernumerary axons. The knockdown of Pld1 and Pld2 could be rescued by expressing the corresponding RNAi-resistant constructs, confirming the specificity of the knockdown phenotype (Pld1 rescue: 17% ± 3% supernumerary axons, Pld2 rescue: 19% ± 2%; Figures 2B and 2D).

To confirm that the extension of supernumerary axons after the knockdown results from a reduced activity of Plds, neurons were treated with the isoform-specific inhibitors VU0359595 (Pld1; 20 nM) or VU0364739 (Pld2; 100 nM) and analyzed at 3 d.i.v. (Figures S3A and S3B).^26^^,^^27^ Like the knockdown, the inhibition of Pld1 or Pld2 resulted in the formation of multiple axons (control: 15% ± 1% supernumerary axons, VU0359595: 34% ± 1%, VU0364739: 36% ± 2%; Figure S3B). By contrast, simultaneous inhibition of both Plds blocked axon extension (control: 18% ± 2% neurons without axons, VU0359595 + VU0364739: 51% ± 9%; Figure S3B). These results show that Pld1 and Pld2 are required for axon extension. Suppression of either Pld1 or Pld2 induces supernumerary axons while simultaneous suppression has the opposite effect and blocks axon formation.

### Constitutively active Pld2 blocks axon formation

To test the effect of activating Plds, an inducible heterodimerization system was used. Plds can be constitutively activated by directing them to the plasma membrane.^28^ A truncated Pld2 was fused to DmrC (GFP-Pld2-DmrC) and recruited to the plasma membrane by the rapalog-induced dimerization with membrane-localized myr-mCherry-2xDmrA (Figure 2E).^28^^,^^29^ Transfected neurons were incubated with solvent or dimerizer for 30 min at 1 d.i.v. and analyzed at 3 d.i.v. (Figures 2F and 2G). Recruitment of GFP-Pld2-DmrC by heterodimerization constitutively activates it at recruitment to the plasma membrane where its activity is not regulated like endogenous Pld2 since it lacks the N-terminal membrane-binding domains.^28^ Constitutively active, membrane-localized Pld2 interfered with axon extension and increased the number of unpolarized neurons without an axon (control: 16% ± 3% unpolarized neurons, dimerizer: 33% ± 1%; Figure 2G). Targeting GFP to the membrane by expression of GFP-DmrC and myr-mCherry-2x-DmrA as a control did not affect axon extension. An inactive Pld2 construct with a mutation in the catalytic domain also did not have an effect (control: 15% ± 4% unpolarized neurons, dimerizer: 15% ± 2%). The expression of the corresponding construct for Pld1 was detrimental to neuronal survival, preventing an analysis of its effects. Taken together, these results indicate that Pld2 plays an important role in regulating axon formation. Increased activity of Pld2 blocks axon formation while reduced Pld2 allows the extension of supernumerary axons.

### Pld2 acts upstream of Pip5ks

**Figure 3:**
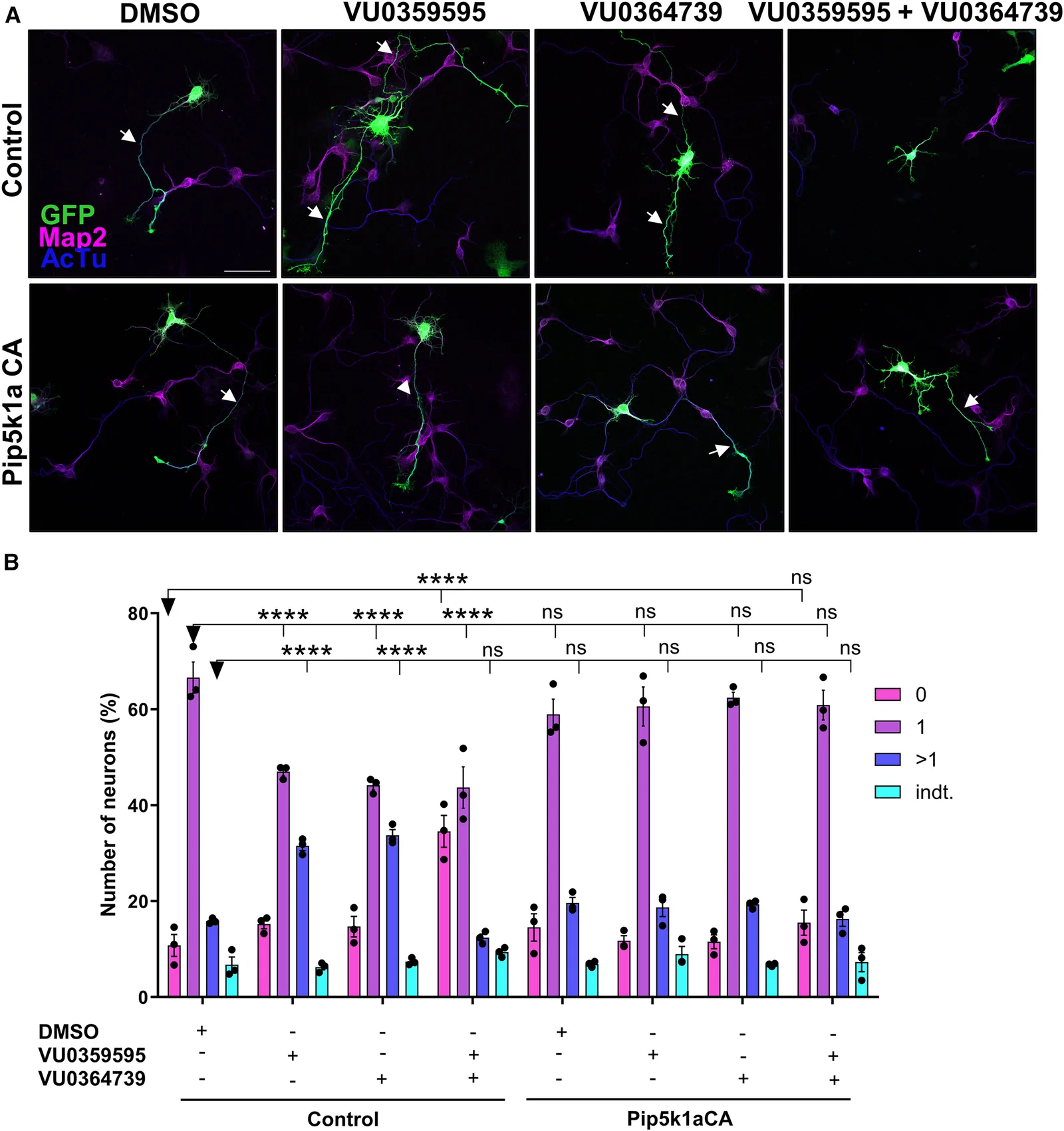
Pld2 acts upstream of Pip5ks (A) Hippocampal neurons were transfected with an expression vector for constitutively active Pip5k1α (Pip5k1a-CA). Inhibitors specific for Pld1 (20 nM of VU0359595) or Pld2 (100 nM of VU0364739) were added at 0 d.i.v. for 72 h and axon formation was analyzed at 3 d.i.v. by staining with anti-acetylated tubulin (blue) and anti-Map2 (magenta) antibodies. Axons are marked by arrows. Scale bars, 50 μm. (B) The percentage of neurons without axons (0, magenta), with a single axon (1, purple), with supernumerary axons (>1, blue) and with indeterminate axons positive for both acetylated tubulin and Map2 (indt., cyan) was quantified for (B). See also Figure S4. Values are means ± SEM from *n* = 3 independent experiments that are biological replicates. (B) Two-way ANOVA followed by Tukey’s post hoc test. *p* > 0.05; ∗*p* ≤ 0.05; ∗∗*p* ≤ 0.01; ∗∗∗*p* ≤ 0.001; ∗∗∗∗*p* ≤ 0.0001.

The extension of supernumerary axons after suppression of Pld2 is similar to the effect of suppressing Pip5ks.^13^^,^^30^^,^^31^ Since PA strongly stimulates the activity of Pip5ks,^15^^,^^16^ we tested if an increase in PI(4,5)P_2_ levels can reverse the Pld2 knockdown phenotype. Neurons were transfected with a vector for constitutively active Pip5k1α (Pip5k1aCA),^13^ incubated with Pld inhibitors for 72 h and analyzed at 3 d.i.v. (Figure 3). Expression of active Pip5k1aCA reversed the effects of inhibiting Pld1 or Pld2. The number of neurons with supernumerary axons was decreased and the number with a single axon increased to control levels (DMSO: 16% ± 0.3% supernumerary axons, DMSO + Pip5k1aCA: 20% ± 1%, VU0359595: 31% ± 1%, VU0364739: 34% ± 1%, VU0359595 + Pip5k1aCA: 19% ± 2%, VU0364739 + Pip5k1aCA: 19% ± 0.5%; Figure 3B). These results indicate that Pld1 and Pld2 act upstream of Pip5ks.

Since active Pip5k1α rescues the effect of reducing membrane tension by a shift to hyperosmotic medium,^13^ we tested if constitutively active Pld2 can also reverse this effect. Pld2 recruitment to the plasma membrane was triggered by addition of dimerizer at 1 d.i.v. in neurons transfected with vectors for myr-mCherry-2xDmrA and EGFP-CA-Pld2-DmrC (Figures S4A–S4D). Subsequently, hyperosmotic medium (neurobasal medium supplemented with 50 mM sucrose) (Figures S4A and S4B) or neurobasal medium containing 5 μM palmitoyl carnitine (PalmC) (Figures S4C and S4D) was applied to reduce membrane tension.^13^^,^^21^^,^^32^^,^^33^^,^^34^^,^^35^^,^^36^ PalmC reduces membrane tension by inserting into the plasma membrane.^33^^,^^34^ Both treatments induced the formation of supernumerary axons. The number of neurons with multiple axons increased from 17% ± 2% in controls to 34% ± 1% in hyperosmotic medium and from 15% ± 1% to 44% ± 3% after treatment with PalmC. This effect was reversed by the activation of EGFP-CA-Pld2-DmrC by dimerizer that reduced the number of neurons with multiple axons (solvent + sucrose: 34% ± 1% supernumerary axons, dimerizer + sucrose: 19% ± 2%, solvent + PalmC: 44% ± 3%, dimerizer + PalmC: 10% ± 3%; Figures S4B and S4D). As a result, the majority of neurons extended a single axon (solvent + sucrose: 51% ± 1% single axon, dimerizer + sucrose: 64% ± 4%). The effect of PalmC could also be reversed by active Pip5k1aCA (control + PalmC: 46% ± 1% supernumerary axons, Pip5k1aCA, + PalmC: 17% ± 2.5%: Figure S4F) as described earlier for hyperosmotic medium.^13^ These results indicate that Pld2 acts upstream of Pip5ks to mediate the effect of reduced membrane tension on axon formation.

### The effect of high viscosity is reversed by activating the Pld2/Pip5k pathway

**Figure 4:**
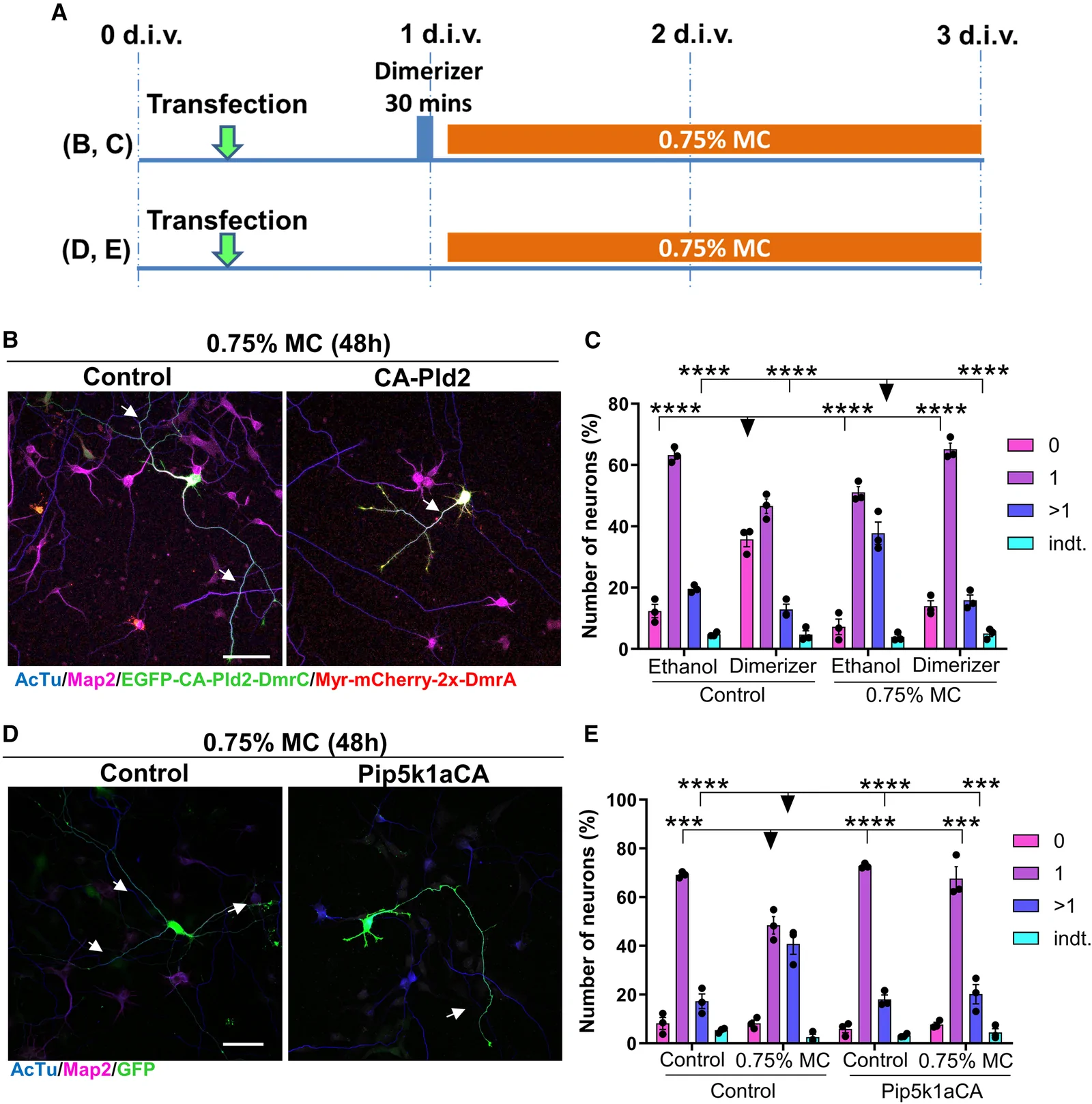
Active Pld2 and Pip5k1α reverse the effect of high viscosity (A) Timeline for the shift to high viscosity. (B) Cortical neurons were transfected with vectors for EGFP-CA-Pld2-DmrC (green) and myr-mCherry-2x-DmrA (red). Solvent (control: ethanol) or dimerizer was added at 1 d.i.v. for 30 min. Subsequently, neurons were cultured in medium supplemented with 0.75% MC for 48 h. Neurons were analyzed at 3 d.i.v. by staining with anti-acetylated tubulin (blue) and anti-Map2 (magenta) antibodies. Axons are demarcated by arrows. Scale bars, 50 μm. (C) The percentage of unpolarized neurons without an axon (0, magenta), polarized neurons with a single axon (1, purple) and neurons with supernumerary axons (>1, blue) or indeterminate neurites (indt., cyan) was quantified for (B). (D) Hippocampal neurons were transfected with an expression vector for constitutively active Pip5k1aCA and cultured with medium supplemented with 0.75% MC starting at 1 d.i.v. for 48 h as indicated. Neurons were analyzed at 3 d.i.v. as in (B). (E) Axon formation was quantified for (D) as in (C). See also Figure S5. Values are means ± SEM from *n* = 3 independent experiments that are biological replicates. (C and E) Two-way ANOVA followed by Tukey’s post hoc test., *p* > 0.05; ∗*p* ≤ 0.05; ∗∗*p* ≤ 0.01; ∗∗∗*p* ≤ 0.001; ∗∗∗∗*p* ≤ 0.0001.

To determine whether the Pld2/Pip5k pathway is involved in the response to increased viscosity, we tested if activation of Pld2 can prevent the induction of supernumerary axons. Neurons were co-transfected with vectors for EGFP-CA-Pld2-DmrC and myr-mCherry-2x-DmrA. After solvent or dimerizer were added at 1 d.i.v. for 30 min to activate Pld2, the cultures were shifted to medium with 0.75% MC and analyzed at 3 d.i.v. (Figure 4). The expression of active Pld2 reversed the effects of high viscosity as the percentage of neurons with supernumerary axons decreased (solvent +0.75% MC: 38% ± 4% supernumerary axons, dimerizer +0.75% MC: 16% ± 2%; Figures 4B and 4C).

Since Pld2 acts upstream of Pip5ks, we tested if active Pip5k1α also reverses the induction of supernumerary axons by MC. Neurons were transfected with a vector for Pip5k1aCA and shifted to high viscosity medium. Expression of Pip5k1aCA, was able to prevent the effects of high viscosity. It reduced the percentage of neurons with supernumerary axons and restored the percentage of neurons with a single axon to a value similar to controls (control: 17% ± 3% supernumerary axons, 0.75% MC: 41% ± 4%, 0.75% MC + Pip5k1aCA: 20% ± 4%; Figures 4D and 4E). These results suggest that the Pld2/Pip5k pathway is involved in the response to high viscosity.

### VLCFAs and glycolipids are required for the response to high viscosity

**Figure 5:**
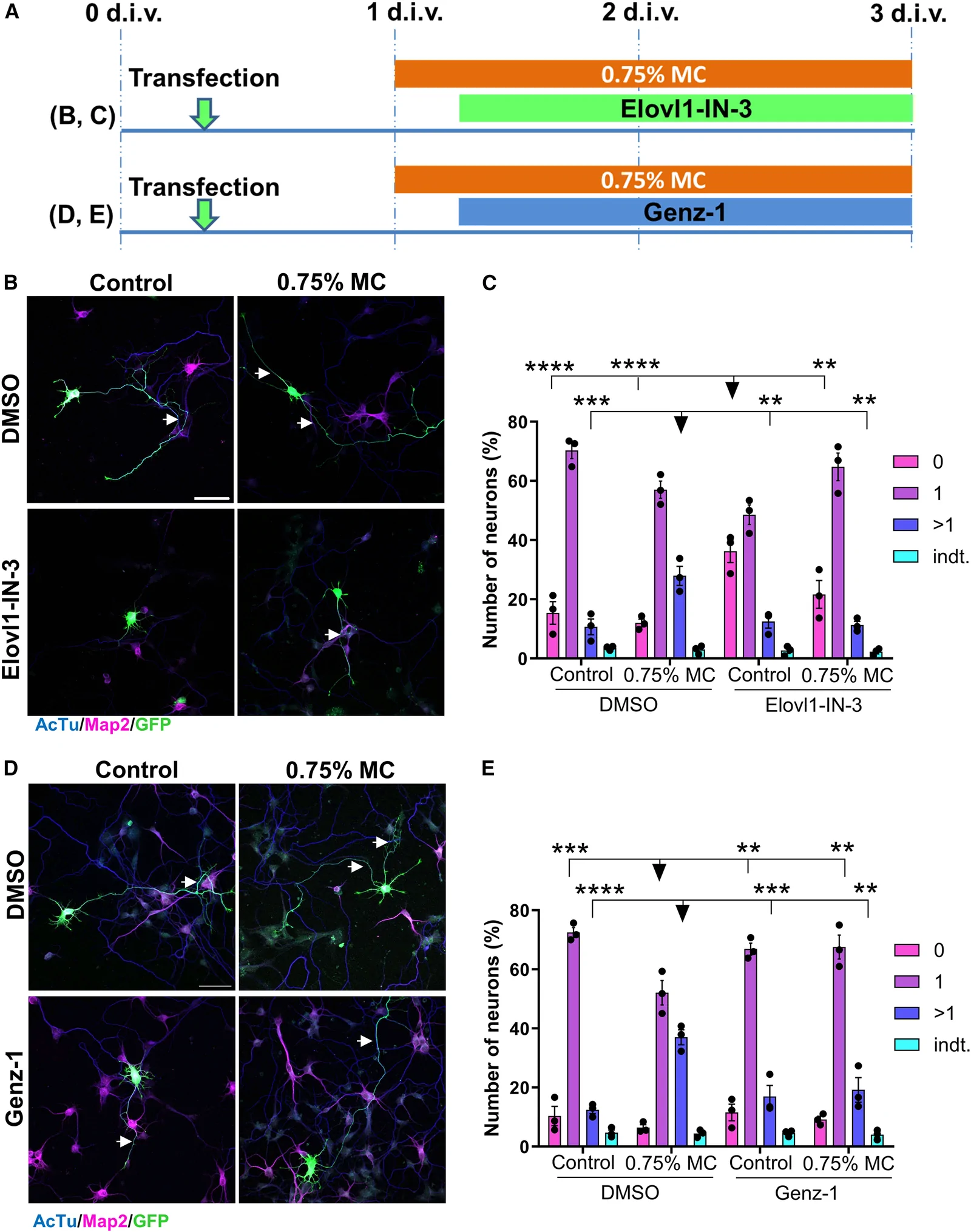
VLCFA and ceramides are required for the response to high viscosity (A) Timeline for addition of inhibitors and the shift to high viscosity. (B) Hippocampal neurons were transfected with an expression vector for GFP (green) and treated with 100 nM Elovl1-IN-3 at 1 d.i.v. for 48 h. Medium (control) or medium supplemented with 0.75% MC and 100 nM Elovl1-IN-3 was added at 1 d.i.v. as indicated in (A). Neurons were analyzed at 3 d.i.v. by staining with anti-acetylated tubulin (blue) and anti-Map2 (magenta) antibodies. Axons are marked by arrows. Scale bars, 50 μm. (C) The percentage of unpolarized neurons without an axon (0, magenta), polarized neurons with a single axon (1, purple) and neurons with supernumerary axons (>1, blue) or indeterminate neurites (indt., cyan) was quantified for (B). (D) Hippocampal neurons were transfected with an expression vector for GFP and treated with 100 nM Genz-1 at 1 d.i.v. for 48 h. Normal medium (control) or medium supplemented with 0.75% MC and 100 nM Genz-1 was added at 1 d.i.v. as indicated in (A). Neurons were analyzed at 3 d.i.v. as in (B). (E) Axon extension was quantified for (D) as in (C). See also Figure S6. Values are means ± SEM from *n* = 3 independent experiments that are biological replicates. (C and E) Two-way ANOVA followed by Tukey’s post hoc test. *p* > 0.05; ∗*p* ≤ 0.05; ∗∗*p* ≤ 0.01; ∗∗∗*p* ≤ 0.001; ∗∗∗∗*p* ≤ 0.0001.

Previous work showed that mechanical force regulates Pld2 by modulating its association with GM1-containing lipid domains.^18^ VLCFAs are a characteristic component of these domains and play an important role in axon extension.^37^ To test if the effect of high viscosity is mediated by VLCFA-containing gangliosides, we targeted the synthesis of VLCFAs and GM1.^34^ The rate-limiting enzyme for VLCFAs synthesis, fatty acid elongase 1 (Elovl1), was inhibited with Elovl1-IN-3.^38^^,^^39^ Addition of 100 nM Elovl1-IN-3 at 1 d.i.v. blocked axon formation and increased the number of unpolarized neurons without an axon (control: 15% ± 4% unpolarized, Elovl1-IN-3: 36% ± 4%; Figures 5A–5C). This result is consistent with a previous report that knockdown of Elovl1 blocks axon formation.^37^ Next, we tested if VLCFAs are required for the extension of supernumerary axons in response to high viscosity. When neurons were shifted to medium with 0.75% MC at 1 d.i.v. in the presence of 100 nM Elovl1-IN-3, the induction of supernumerary axons was blocked. The percentage of neurons with multiple axons was reduced (control: 11% ± 3% supernumerary axons, MC: 28% ± 3%, MC + Elovl1-IN-3: 11% ± 1%; Figures 5A–5C) and those with a single axon increased to a value comparable to controls (control: 70% ± 3% with a single axon, MC: 57% ± 3%, Elovl1-IN-3: 49% ± 3%, MC + Elovl1-IN-3: 65% ± 5%). Elovl1-IN-3 also reversed the extension of supernumerary axons after a shift to 0.1% high viscosity HA (control: 16% ± 1% supernumerary axons, HA: 30% ± 1.5%, HA + Elovl1-IN-3: 17% ± 2.5%; Figures S5A–S5C). Thus, inhibiting the synthesis of VLCFAs blocked the effect of increased viscosity.

A key enzyme for the synthesis of the ganglioside GM1 is glucosylceramide synthase (Ugcg) that produces a precursor by the transfer of glucose to ceramide.^40^^,^^41^ When neurons were treated with the glucosylceramide synthase inhibitor Genz-123346 (Genz-1, 100 nM) at 1 d.i.v., no significant effect on axon formation was observed in cultures with normal, low viscosity medium (Figures 5A, 5D, and 5E). Thus, the synthesis of glucosylceramides is not required for axon extension itself. However, the induction of supernumerary axons by a shift to high viscosity (0.75% MC) at 1 d.i.v. was blocked in the presence of Genz-1 (MC: 37% ± 2.5% supernumerary axons, MC + Genz-1: 19% ± 4%; Figures 5D and 5E). Genz-1 also blocked the extension of supernumerary axons after a shift to 0.1% high viscosity HA (0.1% HA: 35% ± 3% supernumerary axons, 0.1% HA + Genz-1: 15% ± 5%; Figures S5A, S5D, and S5E). The majority of neurons extended a single axon, indicating that glucosylceramides are required for the response to high viscosity. Taken together, these results show that inhibition of VLCFA or glucosylceramide synthesis can prevent the effect of shifting neurons to high viscosity.

In contrast to high viscosity, Elovl1-IN-3 or Genz-1 did not prevent the formation of supernumerary axons after suppression of Pld2. Neurons were transfected with the Pld2 knockdown vector and treated at 1 d.i.v. with Evol1-IN-3 or Genz-1 (Figure S6). At 3 d.i.v., the percentage of neurons with multiple axons induced by the knockdown of Pld2 was not reduced by Elovl1-IN-3 (Pld2 RNAi + DMSO: 39% ± 1% supernumerary axons, Pld2 RNAi + Elovl1-IN-3: 37% ± 1.5%; Figures S6B and S6C) or Genz-1 (Pld2 RNAi + DMSO: 40% ± 2% supernumerary axons, Pld2 RNAi + Genz-1: 37% ± 5%; Figures S6D and S6E). These results indicate that VLCFAs and glucosylceramides are required for the response to high viscosity but not for the extension of axons per se.

### High viscosity and VLCFAs modulate PI(4,5)P_2_ levels

**Figure 6:**
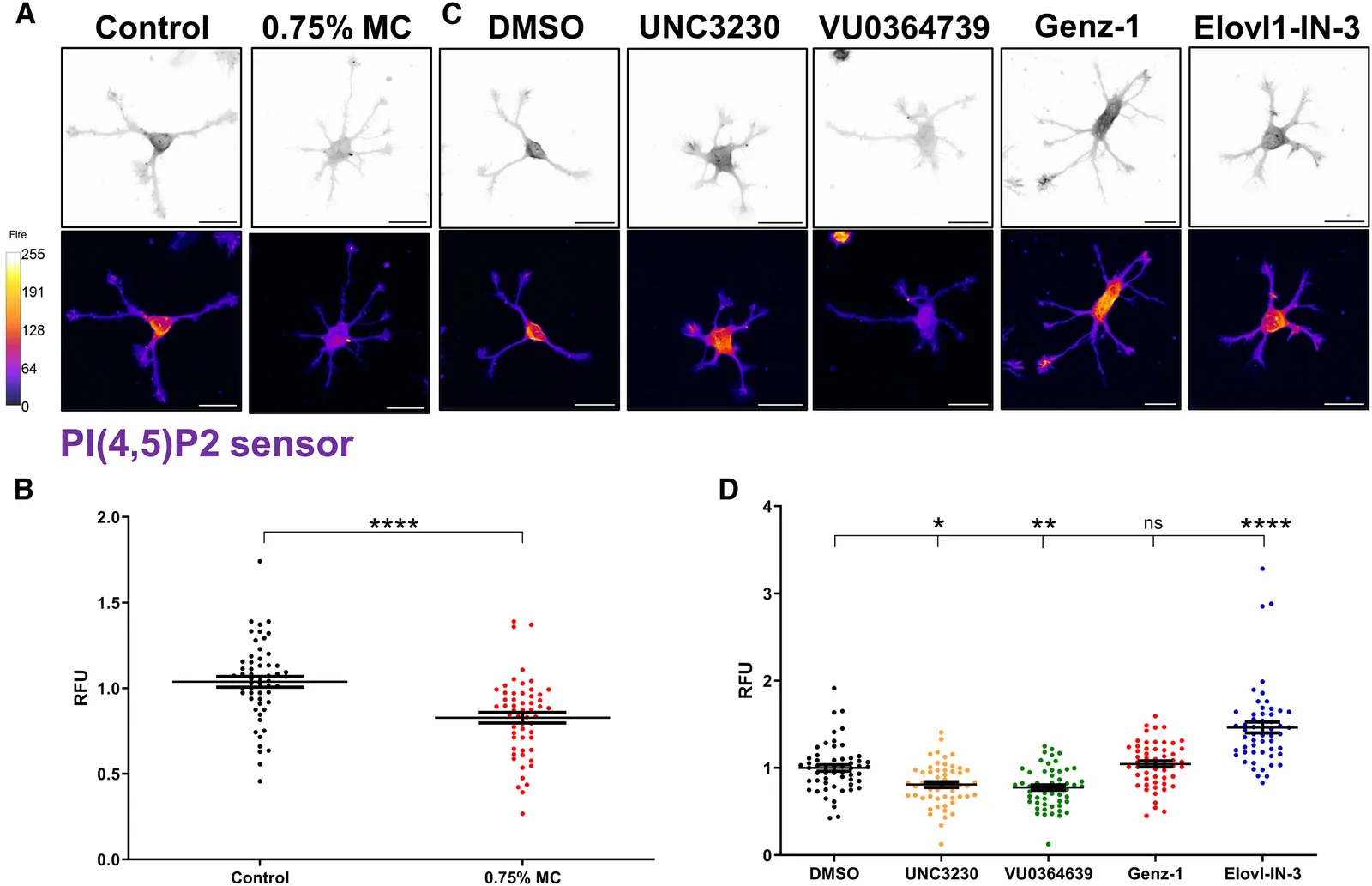
PI(4,5)P_2_ levels are decreased in response to high viscosity (A) Hippocampal neurons were shifted to medium supplemented with 0.75% MC at 1 d.i.v. for 5 h and stained with Alexa Fluor 488 conjugated SNAP-PI(4,5)P_2_ probe. (B) The relative fluorescence intensity per area (RFU, relative fluorescence intensity units) was quantified in neurites for (A). Scale bars, 20 μm. (C) Hippocampal neurons were treated with the indicated inhibitors at 1 d.i.v. for 5 h and stained with the SNAP-PI(4,5)P_2_ probe. (D) The relative fluorescence intensity per area (RFU) was quantified in neurites for (C) Values are means ± SEM from *n* = 3 independent experiments that are biological replicates. (B) Student's *t* test and (D) one-way ANOVA followed by Tukey’s post hoc test. *p* > 0.05; ∗*p* ≤ 0.05; ∗∗*p* ≤ 0.01; ∗∗∗*p* ≤ 0.001; ∗∗∗∗*p* ≤ 0.0001.

Our results indicate that a shift to high viscosity modulates Pld2 to reduce the production of PI(4,5)P_2_ by Pip5ks. To confirm changes in PI(4,5)P_2_, we quantified its levels after a shift to high viscosity at 1 d.i.v. using a recombinant PI(4,5)P_2_ sensor.^42^ Staining with the Alexa Fluor 488-conjugated SNAP-PI(4,5)P_2_ probe revealed a decrease in PI(4,5)P_2_ in neurites in response to high viscosity (Figures 6A and 6B). The reduction of the signal for PI(4,5)P_2_ (control: 1.0 ± 0.03 relative fluorescence intensity unit (RFU), 0.75% MC: 0.8 ± 0.03; Figures 6A and 6B) was similar to the decrease after inhibition of Pip5k1γ or Pld2 (control: 1.0 ± 0.04 RFU, UNC3230: 0.8 ± 0.03, VU0364739: 0.8 ± 0.03; Figures 6C and 6D).

The response to high viscosity depends on glucosylceramides and VLCFAs. Inhibiting glucosylceramide synthesis does not affect axon extension in normal, low viscosity medium, and incubation with Genz-1 also did not change PI(4,5)P_2_ levels (Figures 6C and 6D). VLCFAs are an important component of GM1 domains and their reduction could increase Pld2 activity.^18^^,^^37^ Indeed, inhibition of VLCFA synthesis by Elovl1-IN-3 significantly increased PI(4,5)P_2_ (1.5 ± 0.1 RFU; Figures 6C and 6D), consistent with its role as an inhibitory signal axon extension. These results indicate that increased viscosity and inhibition of VLCFA synthesis modulate PI(4,5)P_2_ production.

### A shift to high viscosity increases GM1 domains

**Figure 7:**
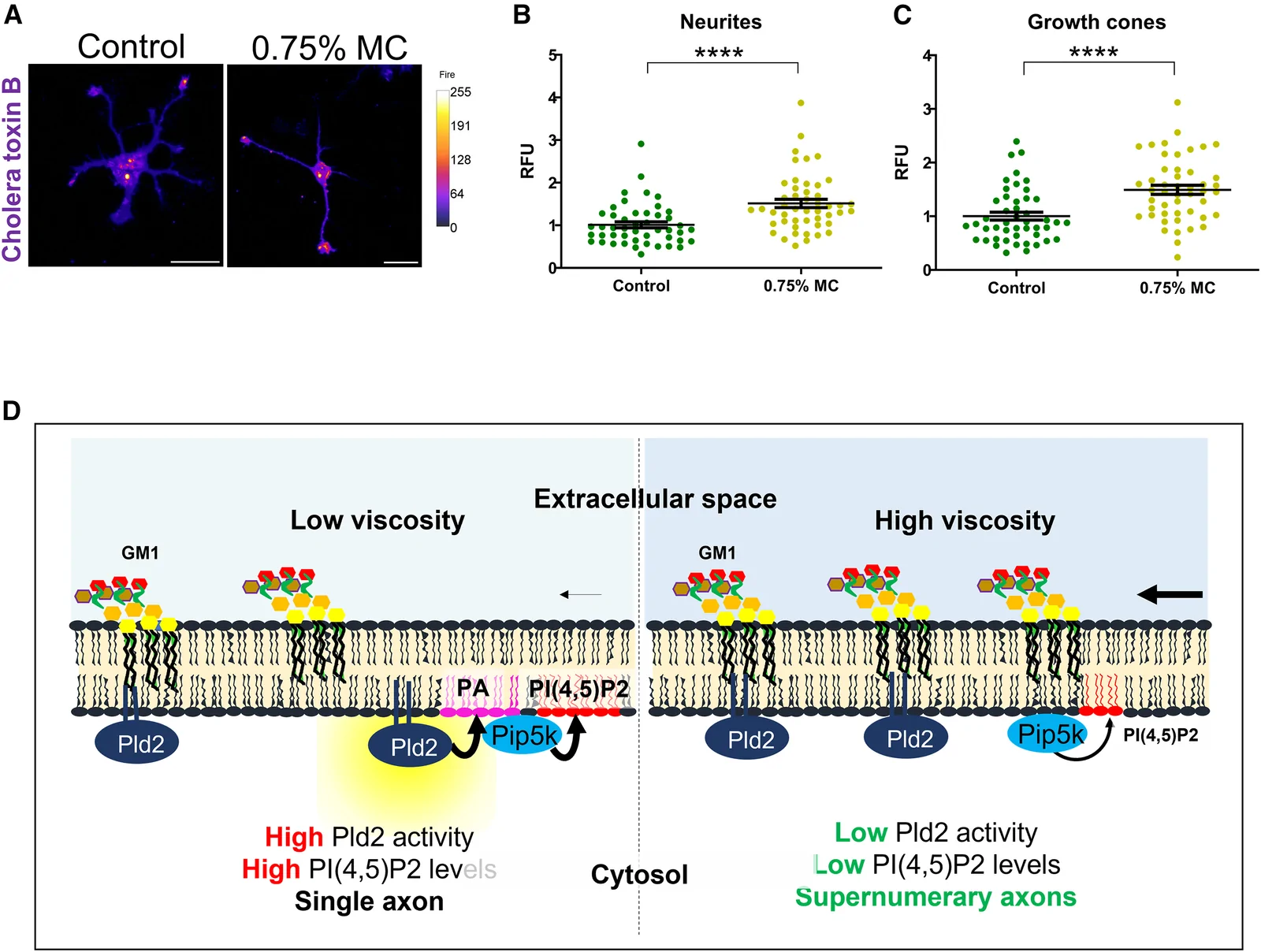
A shift to high viscosity increases GM1 domains (A) Hippocampal neurons were shifted to medium supplemented with 0.75% MC at 1 d.i.v. for 5 h and stained with Alexa Fluor 647 conjugated CtxB. (B and C) The relative fluorescence intensity per area (RFU) was quantified in neurites (B) and growth cones (C) for (A). Scale bars, 20 μm. (D) Unpolarized neurons respond to an increase in extracellular viscosity by extending supernumerary axons. Pld2 acts upstream of Pip5ks to regulate the production of PI(4,5)P_2_ that acts as an inhibitory signal for axon formation, thereby ensuring the formation of a single axon (left). After a shift to high viscosity, an increase in GM1 reduces the activity of Pld2. The lower activity of Pip5ks resulting from a decrease in PA production releases the inhibition of axon formation in minor neurites, which leads to the formation of supernumerary axons (right). Values are means ± SEM from *n* = 3 independent experiments that are biological replicates. (B and C) Student's *t* test: *p* > 0.05; ∗*p* ≤ 0.05; ∗∗*p* ≤ 0.01; ∗∗∗*p* ≤ 0.001; ∗∗∗∗*p* ≤ 0.0001.

Since Pld2 is inhibited by its association with GM1 domains,^18^ high viscosity might modulate Pld2 activity by an effect on GM1. To detect changes in GM1, we used cholera toxin B subunit (CtxB) conjugated to Alexa Fluor 647.^20^^,^^43^^,^^44^ Neurons were exposed to high viscosity medium for 5 h and analyzed immediately before axons start to grow rapidly to exclude possible indirect effects resulting from axon extension for 2 days. Neurons were stained with CtxB after exposure to 0.75% MC for 5 h at 1 d.i.v. and the relative fluorescence intensity was quantified in neurites and growth cones. This revealed an increase in the signal for GM1 in both neurites (control: 1.0 ± 0.1 RFU, MC: 1.5 ± 0.1 RFU; Figures 7A and 7B) and growth cones (control: 1.0 ± 0.1 RFU, MC: 1.5 ± 0.1 RFU; Figures 7A and 7C). Thus, a shift to high viscosity increases GM1 in neurites and growth cones.

## Discussion

Neurons become polarized in the IZ of the developing cortex by extending a single axon.^1^^,^^2^ Several biochemical signals have been identified that promote axon extension but how the physical properties of the extracellular environment affect neuronal differentiation is just beginning to be addressed. The importance of substrate elasticity for neuronal differentiation is now well established.^1^^,^^2^^,^^45^^,^^46^^,^^47^ Our results show that neurons are sensitive to an increase in extracellular viscosity, which induces the extension of supernumerary axons. This response indicates a reduction of an inhibitory signal and depends on plasma membrane glycolipids and Pld2/Pip5k signaling (Figure 7D). We have previously reported that Pip5ks are part of an inhibitory mechanism that ensures the formation of a single axon.^13^ Here we show that Pld2 acts upstream of Pip5ks to restrict the potential of neurites to extend as an axon. High viscosity modulates this inhibitory signal by reducing PI(4,5)P_2_ levels. The induction of supernumerary axons by a shift to high viscosity can be prevented by increasing Pld2/Pip5k signaling and requires VLCFAs and glucosylceramides like GM1 (Figure 7D).

The enzymatic activity of Pip5ks is strongly stimulated by the production of PA^9^^,^^14^ and inhibition of Pld2 reduced the level of PI(4,5)P_2_ in neurites. The phenotypes of suppressing or activating Plds and Pip5ks confirm that Plds function upstream of Pip5ks in neurons. The knockdown or inhibition of either Pld1 or Pld2 results in the formation of supernumerary axons as shown for Pip5ks.^13^ Simultaneous inhibition of both Plds blocks axon extension, indicating a crucial role of PA upstream of PI(4,5)P_2_ synthesis since it is essential for both exo- and endocytosis.^48^^,^^49^^,^^50^ Suppression of either Pld1 or Pld2 reduces the inhibitory effect on axon extension mediated by PA and PI(4,5)P_2_. Simultaneous inhibition of Pld1 and Pld2 is expected to impact processes that require a minimal level of PA/PI(4,5)P_2_ and are essential for membrane trafficking and expansion.^51^^,^^52^ As a consequence, axons cannot extend even in the absence of the inhibitory signal because the processes required for growth are impaired. Constitutively active Pip5k1α rescues the inhibition of Plds, preventing the formation of supernumerary axons. The induction of Pld2 activity at the plasma membrane has the opposite effect and blocks axon formation. Like active Pip5k1α, active Pld2 reverses the effect of reducing membrane tension by exposure to hyperosmotic medium or PalmC. Taken together, these results reveal that Pld2/Pipk1 signaling is an important regulator of axon extension during the establishment of neuronal polarity.

Increasing evidence indicates that changes in the physical properties of the extracellular environment elicit cellular responses by modulating the organization or composition of the plasma membrane.^12^^,^^53^ Our results suggest that Pld2/Pip5k signaling is involved in the response of unpolarized neurons to an increase in viscosity. A shift to high viscosity has the same effect as inhibiting Pld2 or Pip5ks and the activation of either Pld2 or Pip5k1α prevents its effect on axon extension. A possible role of Pld1 in the response to high viscosity could not be analyzed further because activation of Pld1 was detrimental to neuronal survival. It has been previously shown that mechanical force modulates the association of Pld2 with GM1-containing lipid nanodomains and, thereby, regulates its activity.^18^^,^^19^^,^^20^^,^^54^ The importance of GM1 lipid domains for axon extension has been demonstrated by inhibiting the synthesis of VLCFAs that are enriched in these domains.^37^^,^^41^^,^^55^

Our results indicate that gangliosides and VLCFAs are required for the response of neurons to increased viscosity. Inhibiting their synthesis prevents the induction of supernumerary axons by a shift to high viscosity but not after suppression of Pld2. Thus, gangliosides and VLCFAs are not required per se for the extension of axons but for the modulation of Pld2 by extracellular cues. This conclusion is supported by the increase in PI(4,5)P_2_ levels that was observed after inhibition of Elovl1. While axon extension is blocked by Elovl1-IN-3 in normal medium, the inhibitor restores neuronal polarity with a single axon when neurons are shifted to high viscosity medium. Both effects result from the modulation of PI(4,5)P_2_. Inhibition of VLCFA synthesis increases the level of the inhibitory signal PI(4,5)P_2_ while high viscosity has the opposite effect and reduces it.

VLCFAs are an important component of GM1 domains that regulate Pld2 activity.^18^^,^^37^ GM1 in the outer leaflet of the plasma membrane, is required for the response to high viscosity. The extension of a single axon in high viscosity medium in the presence of Elovl-1 indicates that VLCFAs are important for signaling rather than having mainly a structural function. Inhibiting the synthesis of glucosylceramides had no effect on PI(4,5)P_2_ and axon extension in normal, low viscosity medium but prevented the effect of high viscosity. Staining with CtxB revealed a stronger signal for GM1 in neurites after a shift to high viscosity. This increase in GM1 could expand the association with Pld2, which reduces the Pld2-dependent PI(4,5)P_2_ production.^18^

As neurons migrate into the IZ where they extend an axon, they enter an environment with a higher proportion of ECM.^6^ The stiffness of the ECM can affect axon formation in culture and *in vivo*.^46^^,^^56^^,^^57^ There is growing evidence that the viscosity of the medium influences cell migration.^11^^,^^12^ HA as one of the major components would contribute to the viscosity of the ECM as a high MW polymer and its cross-linking by HA-binding proteins.^10^^,^^58^ Shifting unpolarized neurons to a medium with high viscosity HA increases the probability to extend an axon and induces the formation of supernumerary axons. Medium with MC has a similar effect, indicating that the physical properties of these polymers and not their chemical nature is important for the effect. Taken together, our results suggest that an increase in the viscosity of the extracellular environment regulates axon extension by modulating membrane glycolipids and Pld2/Pip5k1 signaling, thereby reducing an inhibitory signal.

### Limitations of the study

The experiments with cultured neurons allow the manipulation of extracellular viscosity and inhibition of specific pathways in unpolarized neurons. However, it remains to be investigated how physical and biochemical signals interact in the developing nervous system. It will be important to quantify the differences in the viscosity of the extracellular space in the developing nervous system, which requires new methods due to its small volume. Our results indicate that high viscosity mediates a reduction in Pld2 activity through GM1. However, the precise molecular mechanism remains to be elucidated. The limitations of the available Pld2 antibodies did not allow to directly quantify the co-localization of endogenous Pld2 and GM1 by super-resolution techniques.

## Resource availability

### Lead contact

Requests for further information and resources should be directed to and will be fulfilled by the corresponding author, Andreas W. Püschel (apuschel@uni-muenster.de).

### Materials availability

All unique/stable reagents generated in this study are available from the lead contact without restriction.

### Data and code availability

• Original western blot images and microscopy data have been deposited at Mendeley Data: https://doi.org/10.17632/2jy4hjvdsh.1 and are publicly available as of the date of publication.
• This paper does not report original code.
• Any additional information required to reanalyze the data reported in this paper is available from the lead contact upon request.

## Acknowledgments

We thank Maria Wenning and Verena Stegemann for technical assistance, Priyadarshini Ravindran for constructing the myr-mCherry-2xDmrA and GFP-DmrC vectors, Claudia Their and Monika Schönhoff for help with viscosity measurements, and Hannes Maib for providing a detailed protocol for detecting PI(4,5)P_2_. This work was supported by the Deutsche Forschungsgemeinschaft (DFG) through SFB 1348.

## Author contributions

T.K., conceptualization, data curation, formal analysis, investigation, visualization, methodology, writing – original draft, and writing – review and editing; A.W.P., conceptualization, data curation, supervision, funding acquisition, validation, visualization, project administration, and writing – review and editing.

## Declaration of interests

The authors declare no competing interests.

## STAR★Methods

### Key resources table

**Table:** 

REAGENT or RESOURCE	SOURCE	IDENTIFIER
**Antibodies**		

Mouse anti-Acetylated Tubulin	Sigma-Aldrich	Cat# T7451; RRID: AB_609894
Rabbit anti-Map2	Millipore	Cat# AB5622; RRID: AB_91939
Mouse anti-β-III Tubulin (Tuj1)	Biotechne	Cat# MAB1195; RRID: AB_357520
Mouse anti-GFP (GF28R)	Thermo Fisher	Cat# A5-15256; RRID: AB_10979281
Mouse anti-myc (9E10)	Sigma-Aldrich	Cat# MABE282; RRID: AB_11204521
Rabbit Anti-Pld1	ProteinTech	Cat# 18355-1; RRID: AB_2163859
Rabbit Anti-Pld2	Invitrogen	Cat# 104015; RRID: AB_2853346
Mouse Tau-1	Sigma-Aldrich	MAB3420; RRID: AB_3068606
Goat anti-rabbit Alexa Fluor 647	Thermo Fisher	Cat# A-21246; RRID: AB_2535814
Goat anti-rabbit Alexa Fluor 594	Thermo Fisher	Cat# A-11012; RRID: AB_141359
Goat anti-mouse Alexa Fluor 647	Thermo Fisher	Cat# A-21235; RRID: AB_2535804
Goat anti-mouse Alexa Fluor 405	Thermo Fisher	Cat# A-31553; RRID: AB_221604
Anti-mouse HRP	Dianova	Cat# 115-035-003; RRID: AB_10015289
Anti-rabbit HRP	Dianova	111-035-003; RRID: AB_2313567

**Bacterial and virus strains**		

*E. coli* XL1-Blue	Stratagene	N/A
*E. coli* BL21	Novagen	N/A

**Chemicals, peptides, and recombinant proteins**		

Neurobasal Medium (NBM)	Thermo Fisher	21103049
B-27 Supplement	Thermo Fisher	17504044
Opti-MEM	Thermo Fisher	31985047
MEM Eagle	PAN Biotech	6350823
Poly-L-ornithine hydrobromide	Sigma	P3655
VU0359595	Cayman Chemical	Cay10955
VU0364739	Tocris	4171
D(+)-Saccharose	Roth	8890.1
Palmitoyl L-Carnitine	Sigma-Aldrich	61251
Hydroxypropyl-methylcellulose	Sigma-Aldrich	H9262
Hyaluronic Acid, high molecular weight	Sigma-Aldrich	53163
Hyaluronic Acid, low viscosity	Sigma-Aldrich	924474
SNAP-Surface® 488	NEB	S9124S
Cholera Toxin Subunit B, Alexa Fluor™ 647 Conjugate	Thermo Fisher	C34778
Elovl1-IN-3	TargetMol	T60219
Genz-123346	MedChem Express	HY-12744

**Critical commercial assays**		

UptiLight HRP blotting chemiluminescent substrate	Interchim	UP99619A
BLOCK-iT™ Pol II miR RNAi Expression Vector Kit	Invitrogen	K493600
iDimerize Inducible Heterodimer System	Takara Bio	635079

**Deposited data**		

Raw and analyzed data	Mendeley Data	https://doi.org/10.17632/2jy4hjvdsh.1

**Experimental models: Cell lines**		

HEK 293T	MPI für Hirnforschung (Frankfurt)	N/A

**Experimental models: Organisms/strains**		

Rat (Wistar)	Harlan Winkelmann	N/A

**Oligonucleotides**		

See Table S1	This paper	N/A

**Recombinant DNA**		

pCAG-GFP	Matsuda and Cepko^59^	11150, Addgene
pcDNA6.2-GW/EmGFP-miR	Invitrogen	K493600
pWZL-Neo-Myr-Flag-Pip5k1A	Boehm et al.^60^	20580, Addgene
pcDNA6.2-GW/EmGFP-C3G RNAi	Di Meo et al.^13^	N/A
myr-mCherry-2xDmrA	This study	N/A
pHet1	iDimerize Inducible Heterodimer System (Takara Bio)	635067
pHet1-Mem1	iDimerize Inducible Heterodimer System (Takara Bio)	635067
pcDNA6.2-GW/H2B-RFP-miR	Di Meo et al.^13^	N/A
pEGFP_DmrC	This study	N/A
pCMV6-Entry-Pld1	OriGene	MR211495
pCMV6-Entry-Pld2	OriGene	MR211273
pcDNA6.2-GW/Pld2_miR_EmGFP_ORF 903	This study	N/A
pcDNA6.2-GW/Pld2_miR_H2B-RFP_ORF 903	This study	N/A
pcDNA6.2-GW/Pld1_miR_EmGFP_ORF 2453	This study	N/A
pcDNA6.2-GW/Pld1_miR_H2B-RFP_ORF 2453	This study	N/A
pEGFP-CA-Pld2	This study	N/A
pCMV6-Entry-Pld1-res (for ORF 2453 miR)	This study	N/A
pCMV6-Entry-Pld2-res (for ORF 903 miR)	This study	N/A
pEGFP_mut(K758R)-CA-Pld2-DmrC	This study	N/A
p6xHis-SNAP-PI(4,5)P_2_ Probe	Maib et al.^42^	211513, Addgene

**Software and algorithms**		

ImageJ	NIH	RRID: SCR_002074; https://imagej.nih.gov/ij/
Prism 10	GraphPad Prism	Version 10.3.1
ZEN 2.3 (blue edition)	Zeiss	N/A
Image Lab 6.0.1	Bio-Rad	N/A

### Experimental model and study participant details

#### Animals

All animal protocols (81.02.04.2021.A344) were performed in accordance with the guidelines of the North Rhine-Westphalia State Environment Agency (Landesamt für Natur, Umwelt und Verbraucherschutz). Rats (Wistar) were maintained at the animal facility of the Institute for Integrative Cell Biology and Physiology (University of Münster) under standard housing conditions at four to five per cage with a 12 h light/dark cycle (lights on from 07:00 to 19:00 h) at constant temperature (23°C) with continuous access to food and water. Timed-pregnant rats were set up in house. Pregnant rats were anesthetized by exposure to isoflurane followed by decapitation and primary cultures prepared from embryos at embryonic day 18 (E18). During dissection, neurons from all embryos (regardless of sex) were pooled. Therefore, any influence of sex on the results of the study cannot be determined.

### Method details

#### Culture and transfection of HEK293T cells

HEK293T cells were cultured in complete MEM medium (1x MEM, 10% fetal bovine serum, 2 mM L-Glutamine, 0.05 mg/mL of Gentamicin) in a humidified incubator at 5% CO2 and 37°C. For transfection, cells were seeded at 2.5 x 10^6^ cells per 10 cm dish one day prior to transfection. Cells were transfected to express recombinant proteins by calcium phosphate precipitation as described previously.^24^

#### Neuronal cell culture and transfection

Primary hippocampal or cortical neurons were obtained from embryonic day 18 (E 18) rat embryos and transfected by calcium phosphate co-precipitation as previously described.^61^ In brief, tissues were dissected, collected in HBSS (Invitrogen) and digested in 0.25% Trypsin/EDTA (Invitrogen) for 8 min. Digested tissue was mechanically dissociated in DMEM (1X DMEM, 2 mM glutamine, 50U/mL gentamycin, 10% fetal calf serum (FCS), Invitrogen) and passed through a cell strainer (40 μm pore size, Sarstedt). Dissociated neurons were seeded onto poly-L-ornithine coated (10 μg/mL, Sigma-Aldrich) coverslips at a density of 40,000 cells/cm^2^. Neurons were allowed to attach for 2 h, when medium was replaced by Neurobasal medium (1x NBM, 2 mM glutamine, 50U/mL gentamycin, 2% B27 (v/v), Invitrogen). For transfection, the culture medium was replaced 3 h after plating by Opti-MEM (Invitrogen) before the transfection. After incubation for 45 min at 37°C at 5% CO_2_, neurons were washed for with Opti-MEM and Neurobasal medium was added. Neurons were transfected at 0 d.i.v. and fixed at 3 d.i.v. if not indicated otherwise.

#### Plasmids

Loss of function experiments were performed with miRNAs generated using the BLOCK-iT Pol II miRNA Expression Vector Kit (Invitrogen). Target sequences were cloned into the pcDNA6.2-GW/EmGFP-miR expression plasmid following the manufacturer’s instructions. The efficiency and specificity of the knockdown was verified by Western blot, immunofluorescence staining and rescue experiments. To generate pcDNA6.2-GW/H2B-mRFP-miR, the sequence for H2B-mRFP was excised from pLV-RFP with DraI/SalI and inserted into pcDNA6.2-GW/EmGFP to replace the EmGFP sequence. Single point mutations were generated by site directed mutagenesis using Phusion High-Fidelity DNA Polymerase (Thermo Fisher). The sequences of the oligonucleotides (Metabion) are listed in Table S1. Pld1 and Pld2 RNAi-resistant constructs were generated from Myc-DDK-tagged-mouse Pld1 (OriGene# MR211495) and Pld2 (OriGene# MR211273). pWZL-Neo-Myr-Flag-Pip5k1A (Pip5k1aCA, Addgene plasmid #20580) was a gift from William Hahn and Jean Zhao, pCAG-GFP was a gift from Connie Cepko (Addgene plasmid #11150), the plasmid for the expression of the 6xHis-SNAP-PI(4,5)P_2_ Probe was a gift from Hannes Maib and David Murray (Addgene plasmid # 211513). The heterodimerization system consisting of the DmrA and DmrC domains (pHet-mem1 and pHet1, Takara Bio) was used for the induced localization of Pld2 to the plasma membrane. The sequence for mCherry was cloned into *p*-Het-Mem1 (DmrA) to generate the expression vector for myr-mCherry-2xDmrA using the primers listed in Table S1. The sequence for EGFP was cloned into pHet1 (DmrC) to express GFP-DmrC. To generate an inducible, constitutively active Plds, the coding sequence of Pld1 (NM_001164056) or Pld2 (BC068317) was fused to EGFP after deleting the N-terminal PX-PH domains (Pld2: aa1-307) by amplifying the relevant sequences using the primers listed in Table S1 and cloning them into pEGFP-C1. The EGFP-Ndel-Pld2 constructs were transferred to *p*-Het-mem1 to generate pEGFP-CA-Pld2-DmrC. The expression vectors for catalytically inactive and Pld2 (K758R) were constructed by side-directed mutagenesis of and pEGFP-CA-Pld2-Dmrc using the primers listed in Table S1.^62^^,^^63^

#### Immunofluorescence staining

Immunofluorescence staining of cultured neurons was carried out as previously described.^61^ The specificity of the anti-Pld1 or anti-Pld2 antibodies was verified by a loss of the signal after knockdown (Figure S2). Neuronal cultures were fixed in 4% paraformaldehyde and 4% sucrose in Phosphate-Buffered Saline (PBS) for 15 min at 37°C and quenched in 50 mM ammonium chloride at room temperature (RT) for 10 min. After three washes with PBS, the cells were permeabilized with 0.1% Triton X-100 for 3 min and incubated in blocking solution (2% normal goat serum, 2% bovine serum albumin and 0.2% cold water fish gelatin Sigma in PBS) for 1 h at RT. Fixed cells were stained overnight with primary antibodies in 10% blocking solution at 4°C. After incubation with the appropriate Alexa-Fluor conjugated secondary antibody (Thermo Fisher) at RT for 2 h, the coverslips were mounted in Mowiol (Sigma-Aldrich).

For the detection of GM1, neurons were fixed in 4% paraformaldehyde, 0.2% glutaraldehyde for 20 min at RT, washed twice in 1x PBS and incubated in 50 mM ammonium chloride at RT for 20 min. Neurons were stained with 1 μg/mL of Cholera toxin B (CTB)-Alexa Fluor 647 (Thermo Fisher) for 1 h in 1× PBS, washed twice in PBS and mounted in Mowiol (Sigma-Aldrich) for further analysis.

#### Purification, conjugation and labeling of recombinant PI(4,5)P_2_ sensor

The recombinant PI(4,5)P_2_ sensor 6xHis-SNAP-PI(4,5)P_2_ Probe was purified as described previously and stored in aliquots at −80°C.^42^ 5 μL of 1 mM SNAP-surface Alexa Fluor 488 was added to 500 μL of purified protein and incubated for 3 h in an overhead shaker at 4°C. Neurons were fixed with 4% paraformaldehyde, 4% sucrose, 0.2% glutaraldehyde in PBS for 20 min and washed twice with PBS. Neurons were incubated with 50 mM ammonium chloride for 20 min and stained with SNAP-surface Alexa 488 conjugated PI(4,5)P_2_ recombinant sensor in blocking solution (5% BSA and 0.5% Saponin in PIPES buffer) for 1 h on ice as described previously.^42^ The stained neurons were fixed in 2% paraformaldehyde, 2% sucrose in PBS on ice for 10 min followed by 10 min at RT. The coverslips were washed with 50 mM ammonium chloride at RT, rinsed with dH_2_O and mounted in Mowiol (Sigma-Aldrich).

#### Viscosity measurement

The viscosity of the medium supplemented with MC was measured using the DMM 2911 densitometer with an oscillating U-tube combined with an Anton Paar micro-viscosimeter (Lovis 2001) at the Institut für Physikalische Chemie, AG Prof. Schönhoff, University of Münster.

#### Western blot

Proteins were separated by SDS-polyacrylamide gel electrophoresis and transferred to polyvinylidene difluoride (PVDF) membranes. Non-specific binding was blocked at RT with 5% non-fat dry milk in Tris-Buffered Saline and 0.1% Tween 20 (TBST) for 1 h. The membrane was incubated overnight with primary antibodies diluted in blocking solution at 4°C. After several washes with TBST, membranes were incubated with HRP-coupled secondary antibodies (Jackson ImmunoResearch Labs) for 2h at RT. Peroxidase activity was visualized by the enhanced chemiluminescence detection system (Interchim) using the ChemiDocTM MP imaging system (Bio-Rad).

#### Image acquisition and analysis

Images were acquired on a Zeiss LSM 700 or Zeiss LSM 800 Airyscan laser scanning confocal microscope with 405, 488, 568 and 647 nm laser lines, and processed using the Zeiss ZEN 2.3 (blue edition) software (Carl Zeiss MicroImaging, Jena, Germany). Neuronal morphology was analyzed using a Plan-Apochromat 40x/1,3 Oil DIC M27 objective.

### Quantification and statistical analysis

#### Quantification

Neuronal polarity was analyzed at 3 d.i.v. as described previously.^24^^,^^61^ Neurons with neurites that were positive for Map2 but negative for acetylated-tubulin were categorized as unpolarized and neurons with a single long and acetylated-tubulin-positive neurite as polarized.^23^ Neurons with several long neurites showing accumulation of acetylated tubulin were counted as neurons with supernumerary axons. Approximately 100 cells per condition were counted for each experiment.

#### Statistical analysis

All plotted values are expressed as means ± standard error of the mean (SEM). All experiments were performed independently at least three times. Specific numbers can be found in the figure legends. If not otherwise indicated, comparisons between two groups were performed using unpaired Student’s t test, comparisons of more than two groups were done using two-way ANOVA followed by Tukey’s test with GraphPad Prism. For all analyses performed, significance was defined as ns: *p* > 0.05; ∗*p* ≤ 0.05; ∗∗*p* ≤ 0.01; ∗∗∗*p* ≤ 0.001; ∗∗∗∗*p* ≤ 0.0001.
