# Supplementary material for: Phospholipase D2 acts upstream of PI4P 5-kinases in the response of neurons to extracellular viscosity
Source: iScience. 2026 Aug 24;29(9):117267. doi: 10.1016/j.isci.2026.117267 (PMC13542513; doi:10.1016/j.isci.2026.117267)
Supplement: Supplementary file 1 [file mmc1.pdf]

iScience, Volume *29*

Supplemental information

Phospholipase D2 acts upstream of PI4P 5-kinases in the response of neurons to extracellular viscosity

Trisha Kundu and Andreas W. Püschel

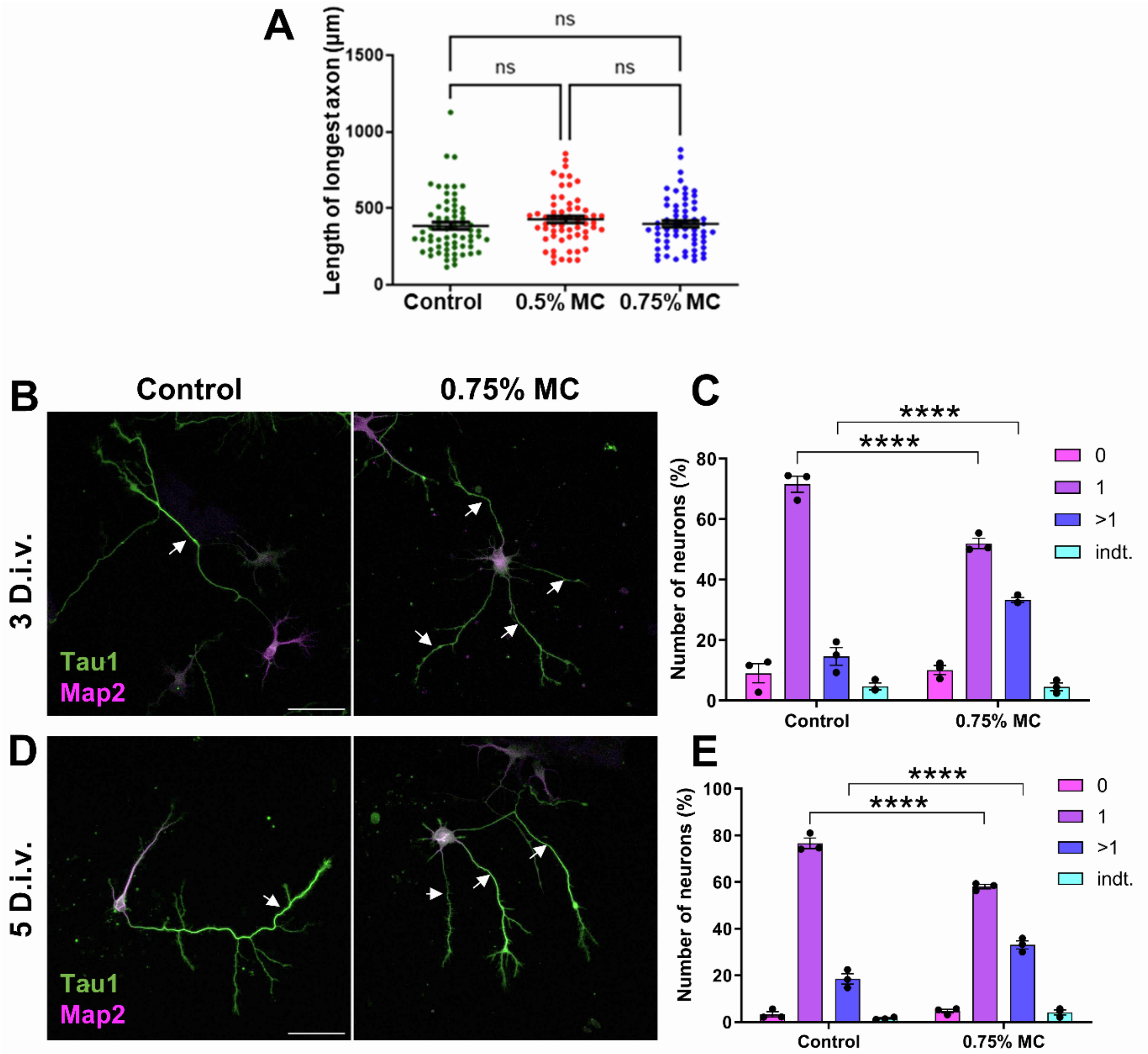

Supplementary Figure S1. A shift to high viscosity induces supernumerary axons but does not change axon length

(A) The length of the longest neurite was quantified for Figure 1E.
(B) Hippocampal neurons were shifted to medium supplemented with 0.75% MC at 1 d.i.v. for 48 hours and analyzed at 3 d.i.v. by staining with Tau-1 (green, axons) and anti-Map2 antibodies (magenta, minor neurites). Axons are marked by arrows. Scale bar: 50µm.
(C) The percentage of unpolarized neurons without an axon (magenta, 0), polarized neurons with a single axon (purple, 1) and neurons with supernumerary axons (blue, >1) or indeterminate neurites (cyan, indt.) was quantified for (B).
(D) Hippocampal neurons were shifted to medium supplemented with 0.75% MC 1 d.i.v. for 96 hours and analyzed at 5 d.i.v. by staining with Tau-1 (green, axons) and anti-Map2 antibodies (magenta, minor neurites). Scale bar: 50µm.
(E) Axon extension was quantified for (D) as in (C).
Values are means ± SEM from n=3 independent experiments that are biological replicates. (A): One-way ANOVA (C and E): Two-way ANOVA followed by Tukey's post hoc test. P > 0.05; *P ≤ 0.05; **P ≤ 0.01; ***P ≤ 0.001; and ****P ≤ 0.0001.

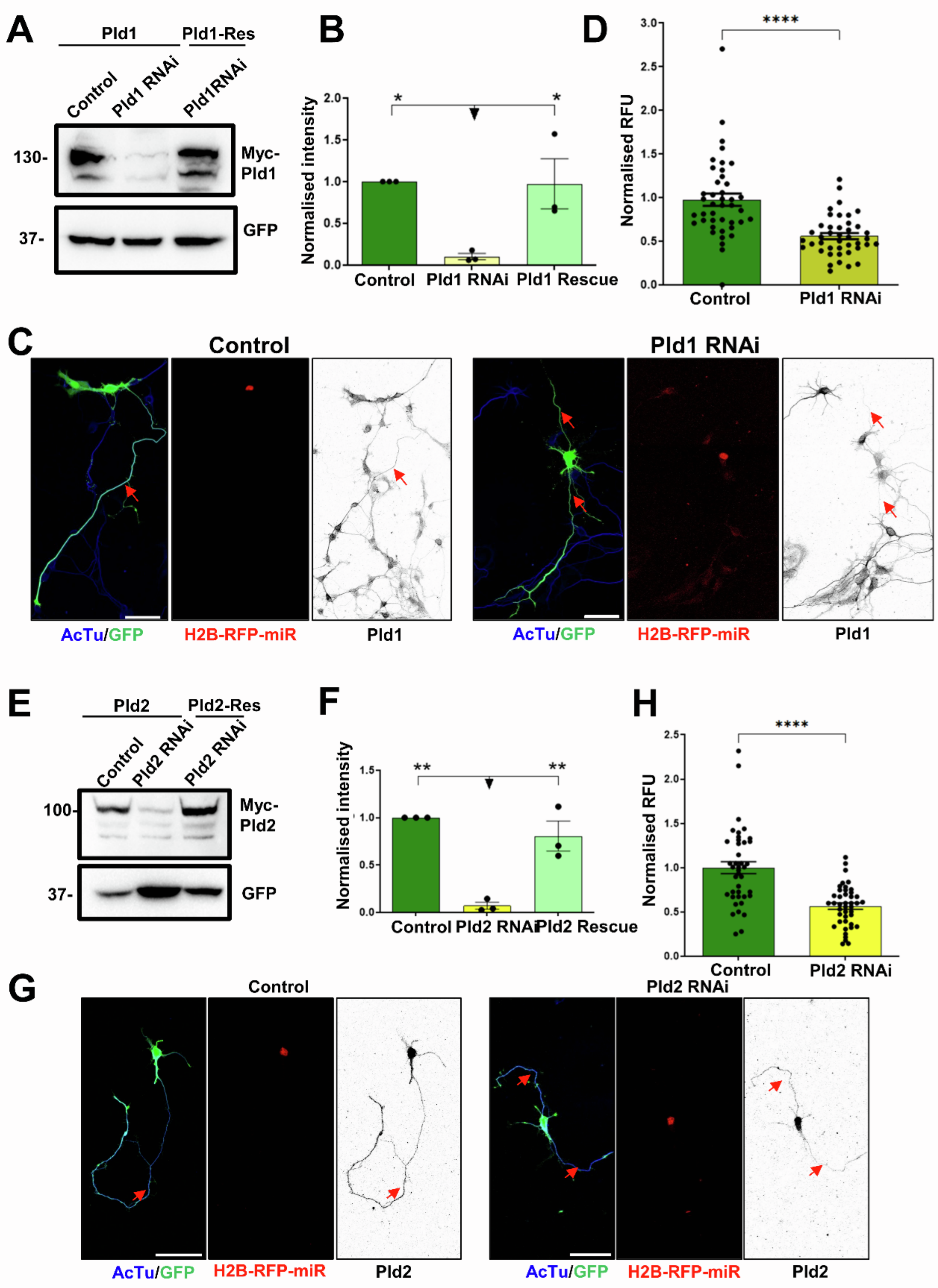
A
Pld1
Pld1-Res
Control
Pld1 RNAi
Pld1RNAi
130-
Myc-
Pld1
37-
GFP
B
Normalised intensity
Control
Pld1 RNAi
Pld1 Rescue
D
Normalised RFU
****
Control
Pld1 RNAi
C
Control
Pld1 RNAi
AcTu/GFP
H2B-RFP-miR
Pld1
E
Pld2
Pld2-Res
Control
Pld2 RNAi
Pld2 RNAi
100-
Myc-
Pld2
37-
GFP
F
Normalised intensity
Control
Pld2 RNAi
Pld2 Rescue
H
Normalised RFU
****
Control
Pld2 RNAi
G
Control
Pld2 RNAi
AcTu/GFP
H2B-RFP-miR
Pld2

Supplementary Figure S2. Validation of knockdown vectors for Pld1 and Pld2

(A) HEK 293T cells were transfected with vectors for myc-Pld1 or RNAi-resistant myc-Pld1-res and control or Pld1 knockdown vectors (Pld1 RNAi) expressing GFP. The expression of Pld1 and GFP was analyzed by Western blot using anti-myc (upper panel) and -GFP (lower panel) antibodies. The molecular weight is indicated in kDa.
(B) The efficiency of the knockdown was quantified for (A) as the relative intensity of the Pld1 signal normalized to the signal for GFP.
(C, D) Hippocampal neurons were transfected with a vector for GFP (green) and a knockdown vector for Pld1 expressing H2B-mRFP to mark transfected cells (red). Neurons were analyzed by staining with anti-acetylated tubulin (blue) and anti-Pld1 (black) antibodies. Scale bar: 50µm.
(E) HEK 293T cells were transfected with vectors for myc-Pld2 or RNAi-resistant myc-Pld2-res and control or Pld2 knockdown vectors (Pld2 RNAi) expressing GFP. The expression of Pld2 and GFP was analyzed by Western blot using anti-myc (upper panel) and -GFP antibodies (lower panel). The molecular weight is indicated in kDa.
(F) The efficiency of the knockdown was quantified for (E) as the relative intensity of the Pld2 signal normalized to the signal for GFP.
(G, H) Hippocampal neurons were transfected with a vector for GFP (green) and a knockdown vector for Pld2 expressing H2B-mRFP to mark transfected cells (red). Neurons were analyzed by staining with anti-acetylated tubulin (blue) and anti-Pld2 (black) antibodies. Scale bar: 50µm.
(H) The relative fluorescence intensity of the signal for Pld2 antibody was quantified for (G).
Values are means ± SEM from n=3 independent experiments that are biological replicates. (B, F): One-way ANOVA followed by Tukey's post hoc test; (D, H): Students t-test. P > 0.05; *P ≤ 0.05; **P ≤ 0.01; ***P ≤ 0.001; and ****P ≤ 0.0001.

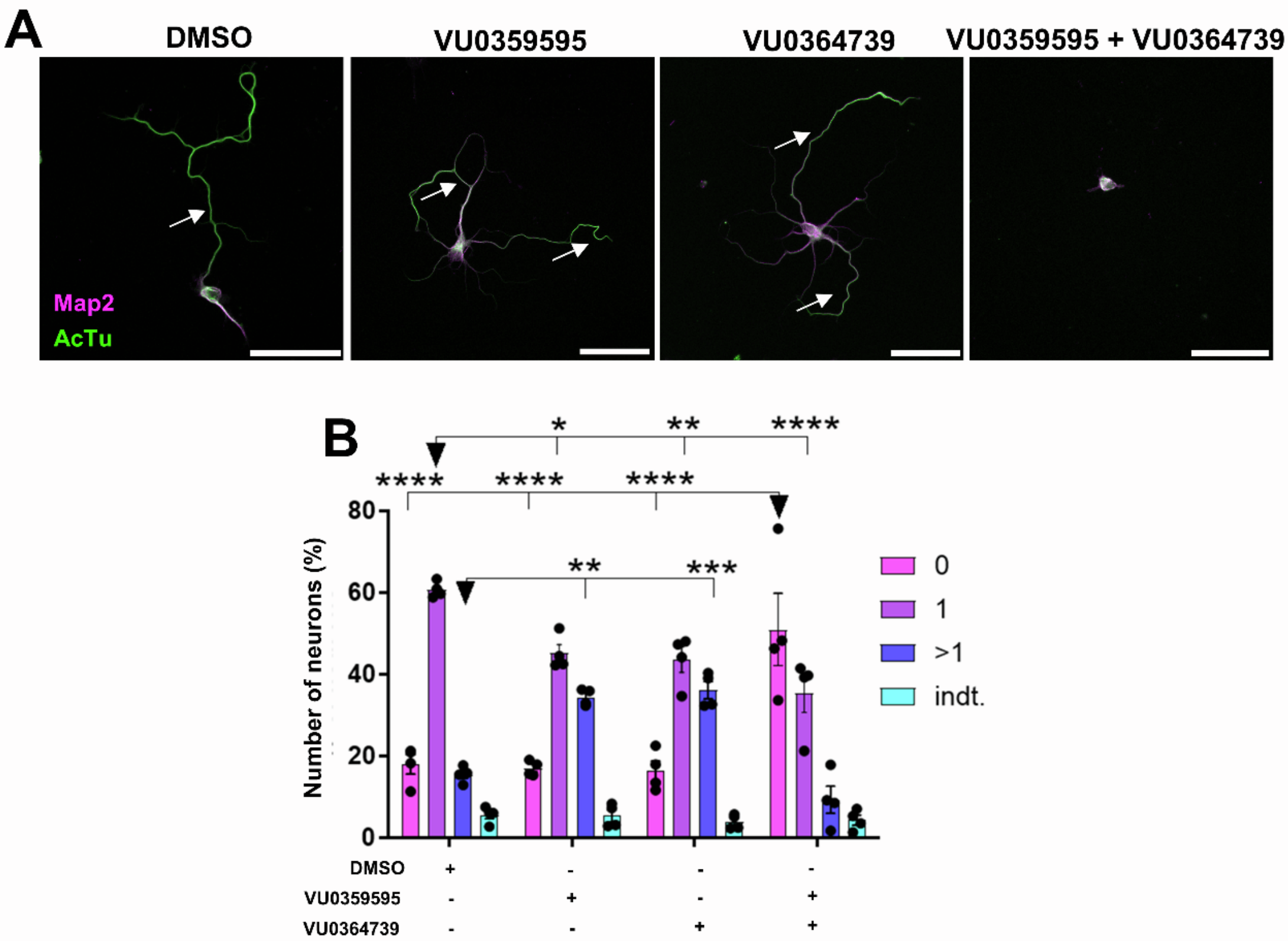

Supplementary Figure S3. Inhibition of Pld1 and Pld2 affects axon extension

(A) Hippocampal neurons were treated at 0 d.i.v. for 72 hours with inhibitors specific for Pld1 (20 nM VU0359595) or Pld2 (100 nM VU0364739). Axon formation was quantified at 3 d.i.v. by staining with anti-acetylated tubulin (green, axonal marker) and anti-Map2 antibodies (magenta, minor neurites).

(B) The percentage of neurons without an axon (0, magenta), with a single axon (1, purple), with supernumerary axons (>1. blue) and with indeterminate axons positive for both acetylated tubulin and Map2 (indt., cyan) was quantified for (A). Axons are marked by arrows. Scale bars: 50µm.

Values are means ± SEM from n=4 independent experiments that are biological replicates. Two-way ANOVA followed by Tukey's post hoc test. P > 0.05; *P ≤ 0.05; **P ≤ 0.01; ***P ≤ 0.001; and ****P ≤ 0.0001.

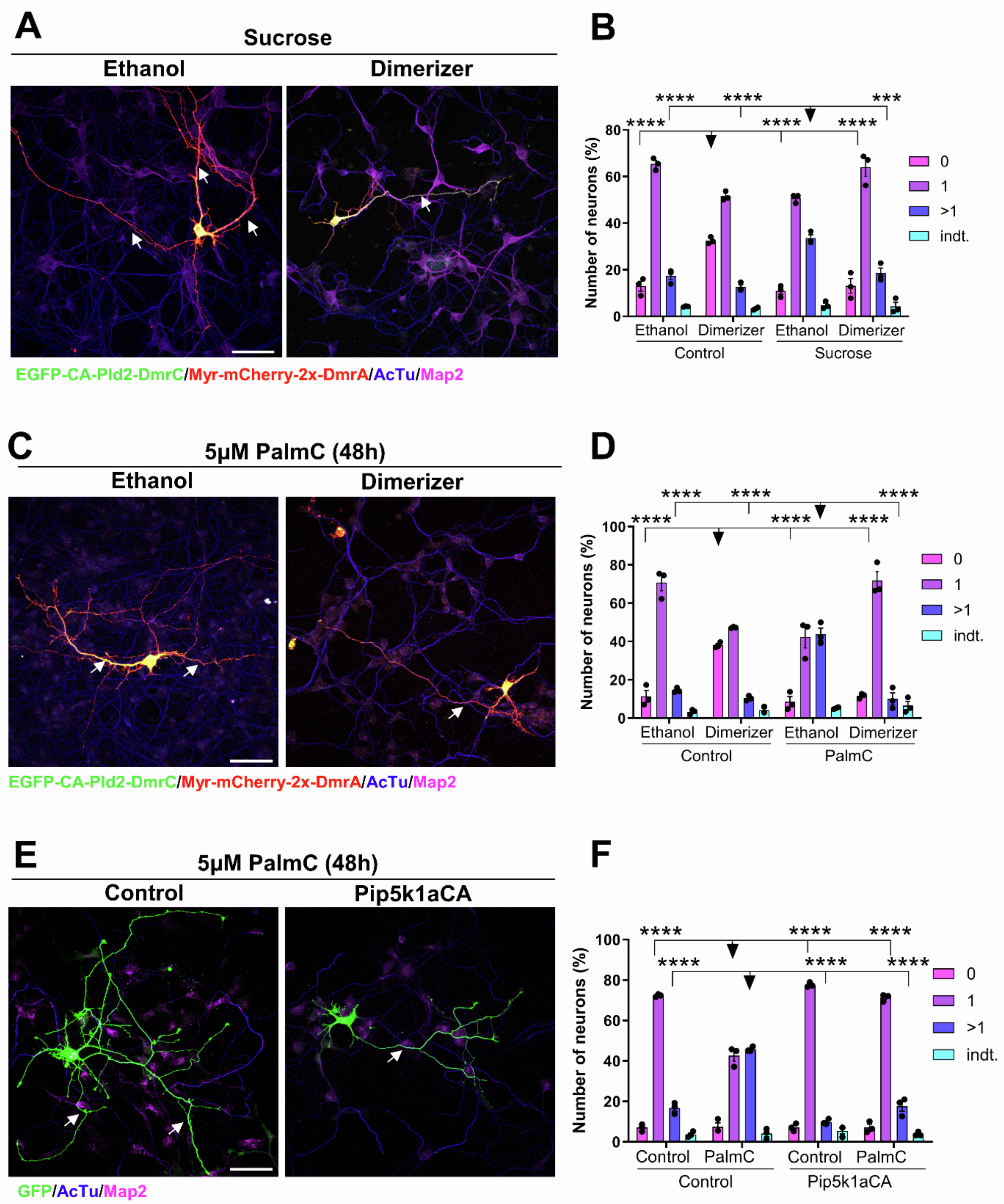
A
Sucrose
Ethanol
Dimerizer
EGFP-CA-Pld2-DmrC/Myr-mCherry-2x-DmrA/AcTu/Map2
B
Number of neurons (%)
0
1
>1
indt.
Ethanol
Dimerizer
Ethanol
Dimerizer
Control
Sucrose
C
5μM PalmC (48h)
Ethanol
Dimerizer
EGFP-CA-Pld2-DmrC/Myr-mCherry-2x-DmrA/AcTu/Map2
D
Number of neurons (%)
Control
PalmC
E
5μM PalmC (48h)
Control
Pip5k1aCA
GFP/AcTu/Map2
F
Number of neurons (%)
Control
PalmC
Control
PalmC
Control
Pip5k1aCA

Supplementary Figure S4. Pld2 acts upstream of Pip5ks

(A) Cortical neurons were transfected with vectors for EGFP-CA-Pld2-DmrC (green) and myr-mCherry-2x-DmrA (red). Solvent (control: ethanol) or dimerizer was added at 1 d.i.v. for 30 minutes. Subsequently, 50 mM sucrose was added for 48 hours and neurons analyzed at 3 d.i.v. by staining with anti-acetylated tubulin (blue) and -Map2 (magenta) antibodies (A). Axons are marked by arrows. Scale bars: 50µm.
(B) The percentage of neurons without an axon (0, red), with a single axon (1), with supernumerary axons (>1) and with indeterminate axons positive for both acetylated tubulin and Map2 (indt.) was quantified for (B).
(C) Cortical neurons were transfected with vectors for EGFP-CA-Pld2-DmrC (green) and myr-mCherry-2x-DmrA (red). Solvent (control: ethanol) or dimerizer was added at 1 d.i.v. for 30 minutes. Subsequently, 5 µM PalmC was added for 48 hours. Neurons were analyzed at 3 d.i.v. by staining with anti-acetylated tubulin (blue) and -Map2 (magenta) antibodies. Scale bars: 50µm.
(D) Axon extension was quantified for (C) as in (B).
(E) Hippocampal neurons were transfected with an expression vector for constitutively active Pip5k1aCA and treated with 5 µM PalmC at 1 d.i.v. for 48 hours. Neurons were analyzed at 3 d.i.v. by staining with anti-acetylated tubulin (blue) and -Map2 (magenta) antibodies. Scale bars: 50µm.
(F) Axon extension was quantified for (E) as in (B).
Values are means ± SEM from n=3 independent experiments that are biological replicates. (B, D, E): Two-way ANOVA followed by Tukey's post hoc test. $P > 0.05$; *$P \leq 0.05$; **$P \leq 0.01$; ***$P \leq 0.001$; and ****$P \leq 0.0001$.

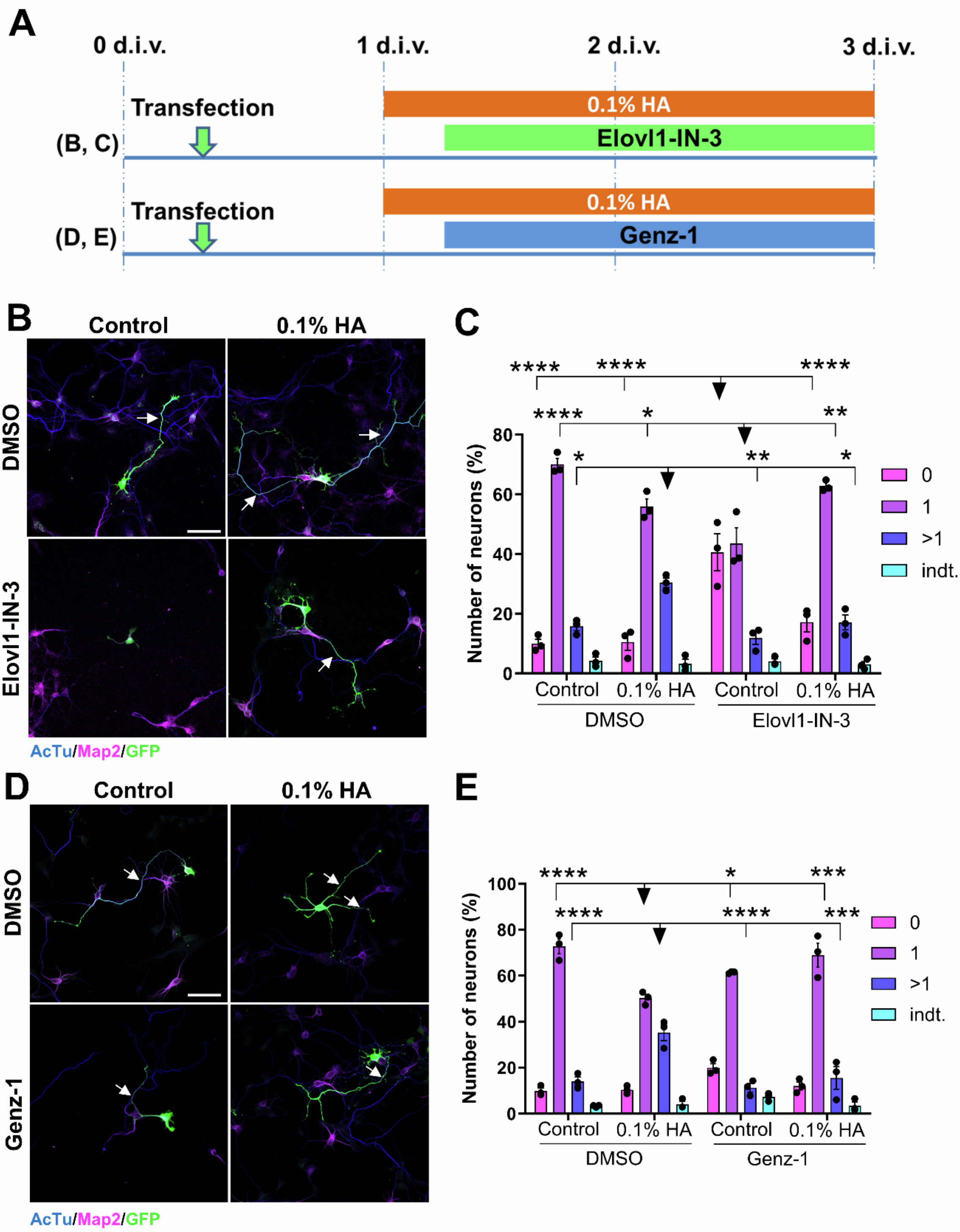
A
0 d.i.v.
1 d.i.v.
2 d.i.v.
3 d.i.v.
Transfection
(B, C)
0.1% HA
Elovl1-IN-3
Transfection
(D, E)
0.1% HA
Genz-1
B
Control
0.1% HA
DMSO
Elovl1-IN-3
AcTu/Map2/GFP
C
Number of neurons (%)
Control
0.1% HA
Control
0.1% HA
DMSO
Elovl1-IN-3
0
1
>1
indt.
D
Control
0.1% HA
DMSO
Genz-1
AcTu/Map2/GFP
E
Number of neurons (%)
Control
0.1% HA
Control
0.1% HA
DMSO
Genz-1
0
1
>1
indt.

Supplementary Figure S5. Active Pld2 and Pip5k1α reverse the effect of high viscosity HA

(A) Timeline for addition of inhibitors and the shift to high viscosity.
(B) Hippocampal neurons were transfected with an expression vector for GFP (green) and treated with 100 nM Elovl1-IN-3 at 1 d.i.v. for 48 hours. Medium (control) or medium supplemented with 0.1% high MW HA and 100 nM Elovl1-IN-3 was added at 1 d.i.v. as indicated in (A). Neurons were analyzed at 3 d.i.v. by staining with anti-acetylated tubulin (blue) and -Map2 (magenta) antibodies.
(C) The percentage of unpolarized neurons without an axon (0), polarized neurons with a single axon (1) and neurons with supernumerary axons (>1) or indeterminate neurites (indt.) was quantified for (B).
(D) Hippocampal neurons were transfected with an expression vector for GFP and treated with 100 nM Genz-1 at 1 d.i.v. for 48 hours. Normal medium (control) or medium supplemented with 0.1% high MW HA and 100 nM Genz-1 was added at 1 d.i.v. as indicated in (A). Neurons were analyzed at 3 d.i.v. as in (C). Axons are marked by arrows. Scale bars: 50µm.
(E) Axon extension was quantified for (D) as in (C).
Values are means ± SEM from n=3 independent experiments that are biological replicates. (C, E): Two-way ANOVA followed by Tukey's post hoc test, $P > 0.05$; $^{*}P \leq 0.05$; $^{**}P \leq 0.01$; $^{***}P \leq 0.001$; and $^{****}P \leq 0.0001$.

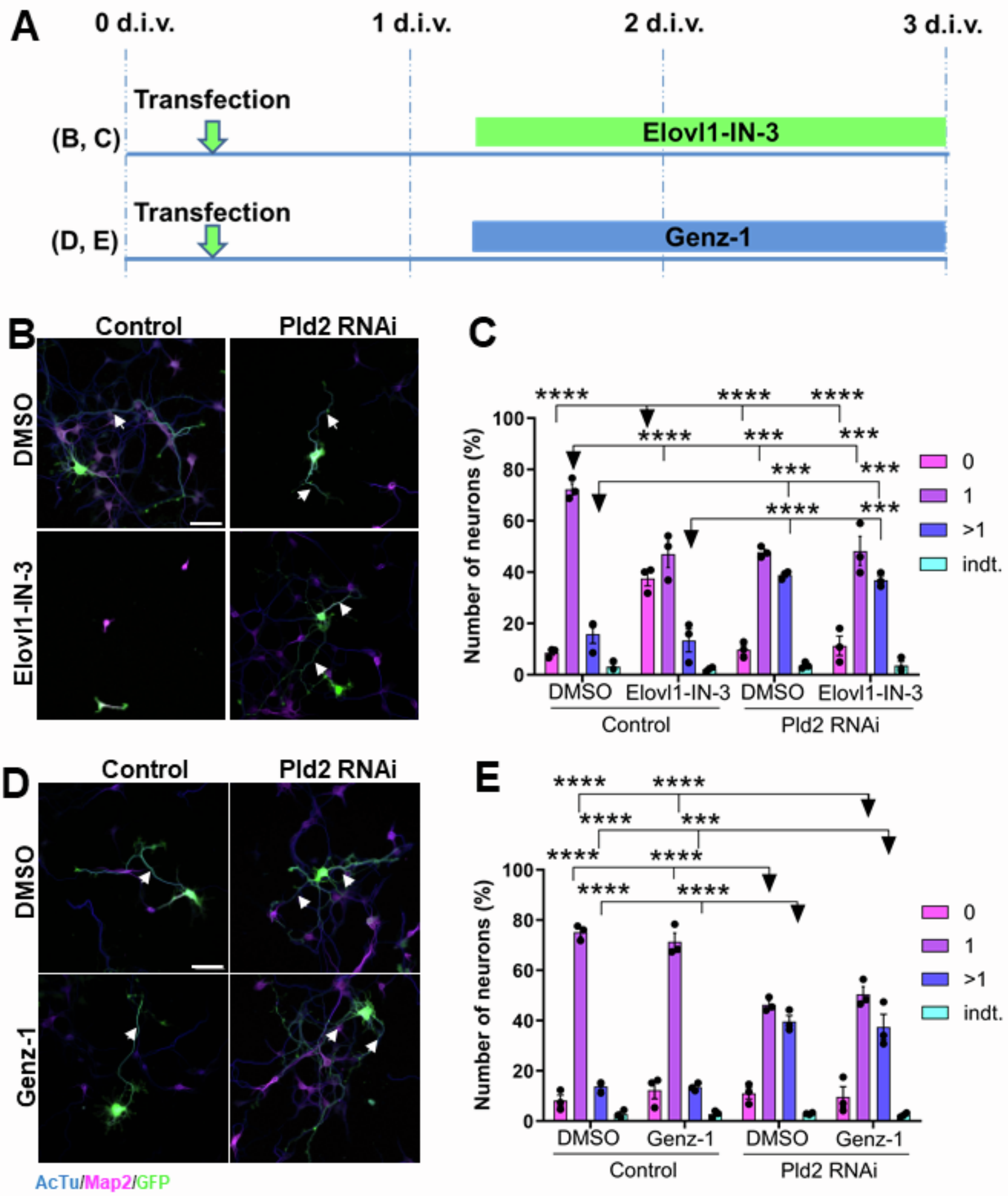
A
0 d.i.v.
1 d.i.v.
2 d.i.v.
3 d.i.v.
Transfection
(B, C)
Elovl1-IN-3
Transfection
(D, E)
Genz-1
B
Control
Pld2 RNAi
DMSO
Elovl1-IN-3
C
Number of neurons (%)
0
1
>1
indt.
DMSO
Elovl1-IN-3
DMSO
Elovl1-IN-3
Control
Pld2 RNAi
D
Control
Pld2 RNAi
DMSO
Genz-1
AcTu/Map2/GFP
E
Number of neurons (%)
0
1
>1
indt.
DMSO
Genz-1
DMSO
Genz-1
Control
Pld2 RNAi

Supplementary Figure S6. Pld2 acts downstream of VLCFA and ceramides

(A) Timeline for the treatment with inhibitors after knockdown of Pld2.
(B and D) Hippocampal neurons were transfected with control or knockdown vectors directed against Pld2 and expressing GFP (green) as indicated and treated at 1 d.i.v. with solvent (control: DMSO), Elovl1-IN-3 (B) or 100 nM Genz-1 (D) for 48 hours. Neurons were analyzed at 3 d.i.v. by staining with anti-acetylated tubulin (blue) and -Map2 (magenta) antibodies. Axons are marked by arrows. Scale bars: 50µm.
(C and E) The percentage of unpolarized neurons without an axon (0), polarized neurons with a single axon (1) and neurons with supernumerary axons (>1) or indeterminate neurites (indt.) was quantified for (B and D).
Values are means ± SEM from n=3 independent experiments that are biological replicates. (C and E): Two-way ANOVA followed by Tukey's post hoc test, P > 0.05; *P ≤ 0.05; **P ≤ 0.01; ***P ≤ 0.001; and ****P ≤ 0.0001.

Data S1: Uncropped Western blot images corresponding to Figure S2.

Figure S2A

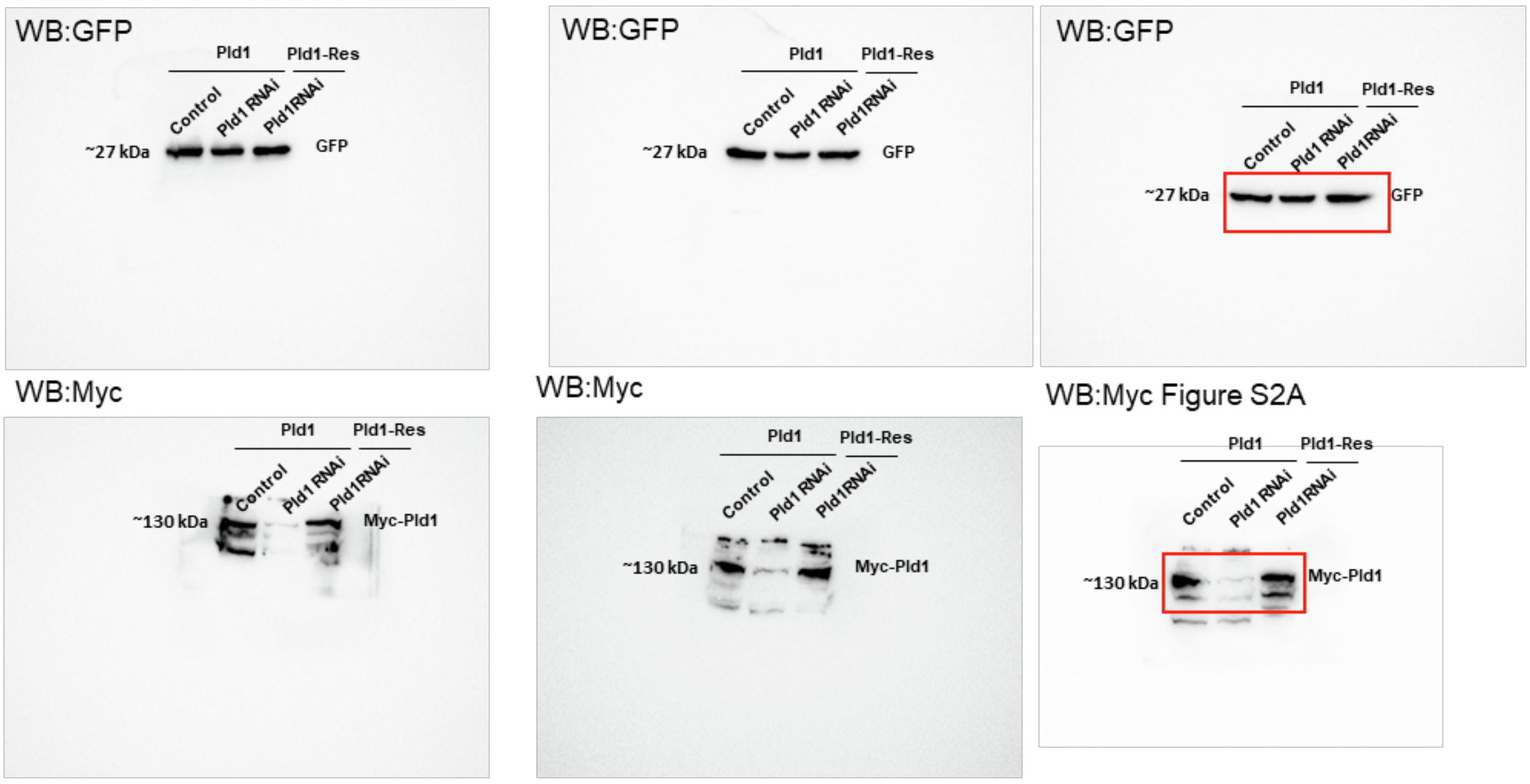

Figure S2E

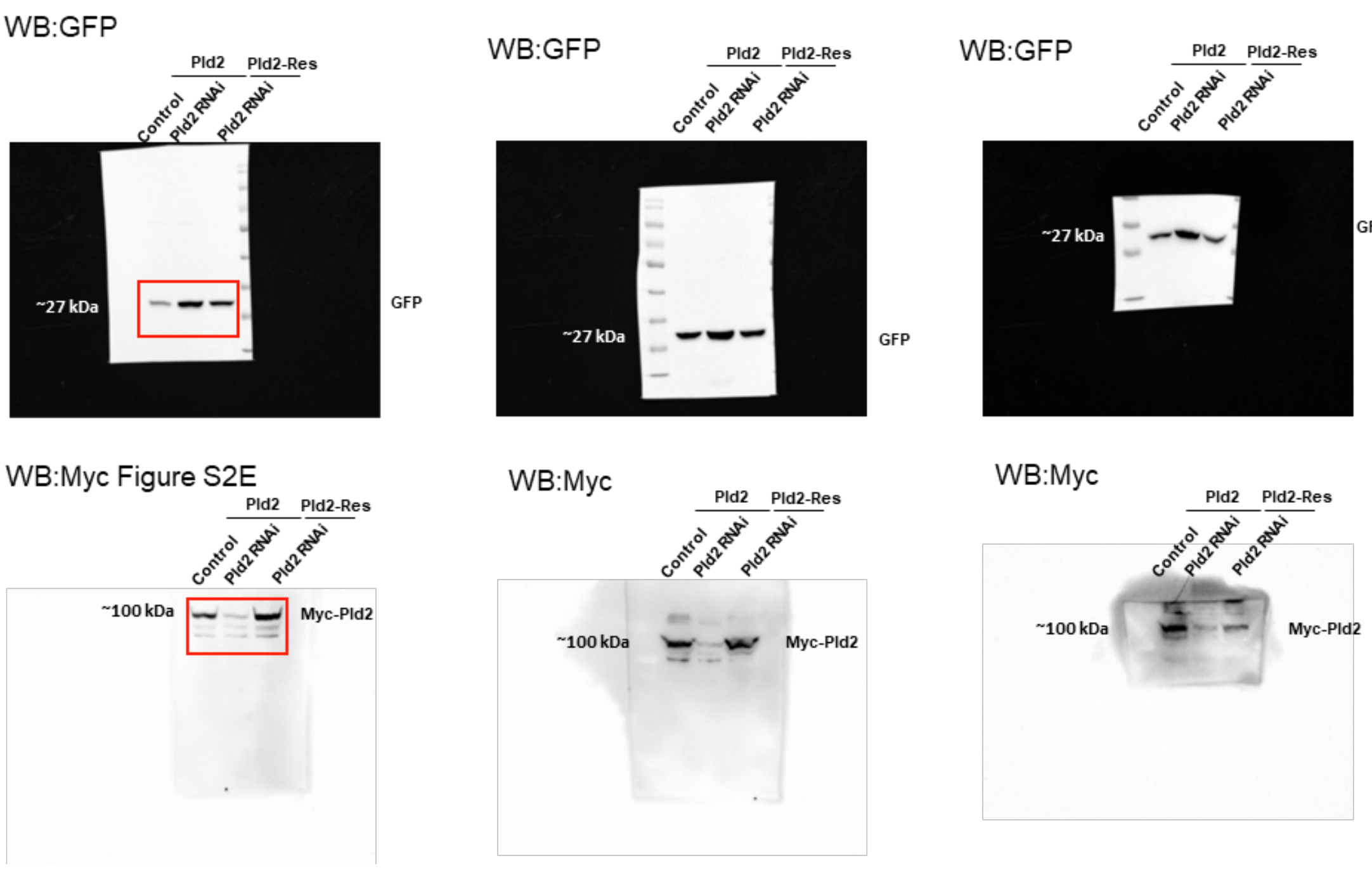

Table S1. Oligonucleotides	
**Primer Name**	**Sequence (5’>3’)**
Pld1 miRNA top strand (ORF 2453)	TGCTGTATAGATGTAGTGCTTGCTGTGTTTTGGCCACTGACTGACACAGCAAGCTACATC
Pld1 miRNA bottom strand (ORF 2453)	CCTGTATAGATGTAGCTTGCTGTGTCAGTCAGTGGCCAAAACACAGCAAGCACTACATCT
Pld2 miRNA top strand (ORF 903)	TGCTGTTGACTGACTTCCAGAAATTCGTTTTGGCCACTGACTGACGAATTTCTAAGTCAG
Pld2 miRNA top strand (ORF 903)	CCTGTTGACTGACTTAGAAATTCGTCAGTCAGTGGCCAAAACGAATTTCTGGAAGTCAGT
Pld1 rescue FP (ORF 2453)	GAGAACAGCAAACATTATATCTATATTGAAAAC
Pld1 rescue RP (ORF 2453)	GTTTTCAATATAGATATAATGTTTGCTGTTCTC
Pld2 rescue FP (ORF 903)	GCCATGACAGAATTCCTCGAGGTCAGTCAACTTTC
Pld2 rescue RP (ORF 903)	GAAAGTTGACTGACCTCGAGGAATTCTGTCATGGC
EGFP_NdeI_Pld2_FP	CCCTCGAGAGGAGCTGGCACAGGGTTC
EGFP_NdeI_Pld2_RP	CCGGATCCGCGGCCGCTTATGTCCACACTTCTAAAGGTATCATC
EGFP-NdeI Pld2 DmrC FP	CCTCTAGAGACGCCACCATGGTGAGCAAG
EGFP-NdeI Pld2 DmrC RP	CCTCTAGATGTCCACACTTCTAAAGGTATCATC
FP DN_PLD2	CATCTATATCCACAGCAGGATGCTCATTGCGGA
RP DN_PLD2	TCCGCAATGAGCATCCTGCTGTGGATATAGATG
FP_eGFP_DmrC	GAACTAGTGTGAGCAAGGGCGAGGAGC
RP_eGFP_DmrC	GGACTAGTCTTGTACAGCTCGTCCATGC
FP_myr-mCherry-2xDmrA	GCTCTAGAATGGTGAGCAAGGGCGAGG
RP_myr-mCherry-2xDmrA	GGACTAGTCTTGTACAGCTCGTCCATGC
